# Ownership Effect Can Be a Result of Other-Derogation: Evidence from Behavioral and Electrophysiological Studies

**DOI:** 10.1371/journal.pone.0166054

**Published:** 2016-11-04

**Authors:** Yunhui Huang, Yin Wu

**Affiliations:** 1 Department of Marketing and Electronic Business, Nanjing University, Nanjing, Jiangsu, China; 2 Research Center for Brain Function and Psychological Science, Shenzhen University, Shenzhen 518060, China; 3 Center for Brain and Cognitive Sciences and School of Psychological and Cognitive Sciences, Peking University, Beijing 100871, China; University of Zurich, SWITZERLAND

## Abstract

Growing evidence suggests that people overvalue their own objects compared to those owned by others, even when the two objects are virtually identical (i.e., ownership effect). Most researchers seem to consider self-enhancement as the underlying mechanism while neglecting the possible process of other-derogation. Here, we attempted to compare these two perspectives, adopting both implicit and neurocognitive methodologies to overcome social desirability confounds. In Study 1, we found that the ownership effect (measured by Implicit Association Test), was correlated with other-derogation but not with self-enhancement (both measured by the Go/No-Go Association Task). In Study 2, by using the event-related potentials (ERPs) technique, we showed that positive-framed other-owned objects elicited significant evaluative incongruity (i.e. indexed by late positive potentials) compared to negative-framed other-owned objects. In contrast, negative-framed self-owned objects did not evoke significant evaluative incongruity relative to positive-framed self-owned objects. Our research suggests that, in addition to the self-enhancement that has been widely demonstrated, it is also important to keep other-derogation in mind when examining the ownership effect.

## Introduction

Imagine two identical coffee mugs: one you own, the other your friend owns. Which is more valuable? From a rational, economic-value perspective, they are equal—made of the same materials, with the same retail value—and are thus effectively interchangeable. However, growing evidence suggests that people will overwhelmingly indicate that their own coffee mug is more valuable than that of their friend. Such overvaluation of self-possessions over other-possessions is known as the ownership effect. Nesselroade, Beggan [1] showed that individuals associated more positive traits with their own possessions than others’ possessions. Similarly, De Dreu and van Knippenberg [2] demonstrated that people set a higher price for self-owned arguments than other-owned arguments, even though the two arguments were quite similar. People also regularly request higher wages for their own time than for another person’s equivalent time [3], suggesting that people even overvalue their own time as compared to that of others. Moreover, behavioral economists have documented the endowment effect—the observation that sellers value their possessions almost twice as much as buyers do, even when the roles of buyers and sellers are arbitrarily assigned [4, 5]. Although the discrepancy between willingness to pay and willingness to accept might be rational because there is a transaction cost, the influence of self-threat on willingness to accept indicates that the endowment effect at least partially involves irrational object evaluation [6].

In order to explain this widely documented ownership effect, most previous research has taken the perspective of self-enhancement [6–8] That is, people have the tendency to self-aggrandize—to hold unrealistic, positive views of the self, exaggerated perceptions of personal control, and unrealistic optimism [9, 10]. Possessions can be viewed as part of the extended self [11] and, because self-evaluation is positively biased [12], people consider self-owned objects as more positive than non-owned or other-owned objects. For example, Beggan [7] demonstrated that individuals rated an object more favorably once they owned it than when they did not own it. Similarly, owners who already possessed a mug were willing to pay more to purchase a second mug than non-owners [8].

However, previous research ignored the potential confound that biased object evaluation may be due to a positively biased view of one’s own objects (self-enhancement) as well as a negatively biased view of other people’s objects (other-derogation). In the present research, we aim to disentangle these two confounding factors by investigating if other-derogation plays a role in self-biased object evaluation.

As we described above, although there is plenty of evidence in support of self-enhancement as a mechanism underlying the ownership effect, the available empirical studies directly associated with other-derogation are inconsistent. Beggan [7] found self-affirmation led to higher object evaluation than self-threat among non-owners. This suggests that people have a stronger tendency to derogate objects owned by others when they feel bad (vs. good) about themselves. If self-enhancement was the only cause of the ownership effect, self-threat/self-affirmation should only take effect on evaluation of self-objects, but not other-objects. Dommer and Swaminathan [6], however, found self-threat did not influence buyers’ willingness-to-buy, which fails to support other-derogation.

Although relatively little attention has been paid to other-derogation in the ownership effect, previous research has demonstrated the important role of other-derogation in social life. Fein and Spencer [13] found people were more likely to derogate a stereotyped target (i.e., gay man) when under self-image threat; furthermore, the degree of derogating a stereotyped target mediated the increase in the evaluators’ self-esteem. Research in social comparison has also suggested that people utilize downward social comparison strategies (e.g., either passively or actively comparing oneself with others who are inferior on some important dimension) to maintain positive well-being [14]. These findings suggest that people can boost their own self-views by viewing others as more negative. That is, not only self-enhancement but also other-derogation can serve the motive to protect the self through the psychological process that “I may not be that good, but others are worse.” We reasoned that it is plausible that other-derogation can go beyond views of others and extend to other-owned objects as well, and thus could drive the ownership effect.

Most of the previous research on the ownership effect asked participants to self-report evaluation about objects either assigned to them or not. If they received the target object as a gift [7], they might suffer from the social desirability to show the appreciation for that gift. If they received the target object for later usage (e.g., an argument) [2], they might be influenced by wishful thinking and rate the target object as more positive [15]. Moreover, participants sometimes “make up” attitudes because they are explicitly asked to self-report and they do not wish to look foolish for not having an attitude or taste [16]. On the other hand, our primary aim in the present study was to provide evidence that other-derogation could serve as the mechanism underlying the ownership effect. It is likely that some participants may consider derogating other-owned objects as socially undesirable.

In order to solve these problems, we adopted implicit measurement and event-related potentials (ERP) methodologies in the present research. In our studies, we asked the participants to imagine a scenario involving both objects they owned and objects owned by others. In this way, we were able to measure the within-subject difference between people’s reaction to self- and other-owned objects. Previous research has demonstrated that imagination is sufficient to elicit relevant attitudes which could be captured by the implicit measures [17, 18]. Moreover, if the self-enhancement and other-derogation effects are obtained with imagined stimuli, they are likely to be even stronger with real stimuli. In recent work, Huang, Wang [19] used the implicit association test (IAT) [20] to study the ownership effect, and found that individuals held a relatively more positive attitude toward self-owned objects (vs. other-owned objects). However, the IAT cannot disentangle the relative contribution of overvaluation of self-possession (i.e., self-enhancement) and devaluation of other-possession (i.e., other-derogation). Thus, we employed the Go/No Go Association Task (GNAT) [21] in Study 1. In Study 2, we used the ERP technique, which has good time resolution, and has been used extensively in recent research on attitudes and social cognition [22, 23].

## Study 1

### Materials and Method

#### Participants

Ninety-two undergraduates in China participated in the experiment for course credit. They first completed the IAT and then the GNAT. The study was conducted in accordance with Declaration of Helsinki and was approved by the Institutional Review Board of the School of Psychological and Cognitive Sciences, Peking University as an exempt research. All data were analyzed anonymously

#### The IAT phase

The IAT experiment used a single-factor design. The independent variable was the perceived ownership of the objects, either self or other. Following the procedures of Huang, Wang [19], participants were asked to imagine a scenario in which six objects (i.e., a mug, a small figurine, chocolate, candy, a pen, and a ruler) were assigned either to them or to an unspecified other person. Participants needed to classify the words shown on the computer screen into two target categories (objects owned by the self vs. objects owned by the other) and two attribute categories (positive vs. negative). Please see Huang, Wang [19] for the detailed items of the categories.

The IAT consisted of five classification tasks. Participants pressed a left key and a right key to classify words shown on the screen into the categories required (please see Table 1 for details). They were required to respond as fast as possible while trying to minimize mistakes. Note that in the initial combined task and the reversed combined task, words might come from both target categories and attribute categories, and participants had to classify them accordingly. Moreover, the initial combined task and the reversed combined task have two blocks each: Block 3/Block6 for practice and Block4/Block 7 for data collection/analysis.

**Table 1:** The Procedure of Implicit Association Test.

Block	Task	Trials	Response key assignment	
			Left key	Right key
1	Attribute discrimination	24	Positive	Negative
2	Initial target discrimination	24	Self-owned	Other-owned
3	Initial combined task	24	Positive; Self-owned	Negative; Other-owned
4	Initial combined task	48	Positive; Self-owned	Negative; Other-owned
5	Reversed target discrimination	48	Other-owned	Self-owned
6	Reversed combined task	24	Positive; Other-owned	Negative; Self-owned
7	Reversed combined task	48	Positive; Other-owned	Negative; Self-owned

Note. Positive = positive adjectives; Negative = negative adjectives; Self-owned = self-owned objects; Other-owned = other owned objects. Participants pressed the left and right key to classify the word that appeared on the screen. The order of the IAT blocks as well as the objects assigned to self-owned versus other-owned were counterbalanced between subjects.

In order to minimize any order effect, trials in each block were randomized. Moreover, half of the participants completed the seven blocks in the order presented in Table 1 and for the other half, Blocks 2, 3, and 4 were swapped with Blocks 5, 6, and 7. Participants’ reaction times for each trial and their error rates were recorded.

The underlying logic of the task is that it would be easier (and thus faster) when the two concepts that share the same response are strongly associated than when they are weakly associated [24]. The error rate was used to exclude participants who did not treat the task seriously enough.

#### The GNAT phase

After the IAT, the participants were asked to complete the GNAT based on the scenario they read before the IAT. Similarly, the GNAT involved four stimulus categories (e.g., self-owned vs. other-possessions, positive vs. negative words). The items representing self-owned and other-owned possessions were the same as the IAT. There were 24 positive words and 24 negative words from Nosek and Banaji [21]. In each trial, specific types of stimuli (e.g., positive words; see Table 2) were assigned as targets; the remaining stimuli served as distractors. Lists of target terms were provided prior to each block, and the labels of the target categories remained in the upper left and right corners. Participants were instructed to press the space bar when a target appeared and to refrain from pressing the space bar when a distractor appeared. Stimuli were presented for 600 ms each. After each trial, a green O (for a correct response) flashed on the screen for 250 ms or a red X (for an incorrect response) flashed on the screen for 450 ms.

**Table 2:** The Procedure of Go/No-Go Association Task.

Blocks	Tasks	Trials	Stimuli
1	Practice	16	Targets: positive; Distractors: negative
2		16	Targets: negative; Distractors: positive
3		16	Targets: self-owned; Distractors: other-owned
4		16	Targets: other-owned; Distractors: self-owned
5	Practice	16	Targets: positive or self-owned; Distractors: negative or other-owned
	Main task 1	60	Targets: positive or self-owned; Distractors: negative or other-owned
6	Practice	16	Targets: negative or self-owned; Distractors: positive or other-owned
	Main task 2	60	Targets: negative or self-owned; Distractors: positive or other-owned
7	Practice	16	Targets: positive or other-owned; Distractors: negative or self-owned
	Main task 3	60	Targets: positive or other-owned; Distractors: negative or self-owned
8	Practice	16	Targets: negative or other-owned; Distractors: positive or self-owned
	Main task 4	60	Targets: negative or other-owned; Distractors: positive or self-owned

Note. Positive = positive adjectives; Negative = negative adjectives; Self-owned = self-owned objects; Other-owned = other owned objects. Words were shown on the screen one-by-one. Participants pressed the space bar for signals, and did not press any key for noises. The order of Blocks 1, 2, 3, and 4 was random, while the order of Blocks 5, 6, 7, and 8 was also random.

As shown in Table 2, the GNAT had eight blocks. Throughout four practice blocks of 16 trials each, participants were asked to simply sort positive/negative words from negative/positive words or self-/other-owned possessions from other-/self-owned possessions. These trials were followed by four complex 76-trial blocks, in which participants had to discriminate among four sets of stimuli (positive words, negative words, self-owned possessions, and other-owned possessions). Among the 76 trials, the first 16 trials were practice trials, which were not included in the data analysis. The order of the complex blocks was counterbalanced across participants.

In each trial, we asked participants to respond to self-possessions and positive words (i.e., targets) but to do nothing in response to other items (i.e., distracters). Errors were compared to a corresponding block of trials in which the participants were asked to respond to self-possessions and negative words. Positive attitudes towards self-possessions (i.e., self-enhancement) would lead to higher accuracy rate in the former block than in the latter one. Following similar procedures, attitudes toward other-possessions can also be measured [21]. For the block of other-possessions, higher accuracy for negative rather than positive trials would indicate other-derogation.

### Results

Main results of the IAT and the GNAT are summarized in Table 3.

**Table 3:** Result Summary of the IAT and the GNAT.

IAT	Self + Positive vs. Other+ Negative	Other + Positive vs. Self + Negative	t-test	*p*	Findings
Latency	821ms (197ms)	949ms (247ms)	Paired t-test using log-transformed data, t(87) = 6.85	1.01E-09	Ownership effect
D-2SD	.30 (.46)		One sample t-test against 0, t(87) = 6.13	2.54E-08	
GNAT	Positive	Negative	Paired t-test	*p*	Findings
Self	1.73 (.93)	1.31 (.71)	*t*(87) = 4.41	2.90E-05	Self-enhancement
Other	.91 (.75)	1.47 (.84)	*t* (87) = 6.42	6.91E-09	Other-derogation

#### The IAT results

Our first goal was to corroborate the ownership effect using the IAT. We analyzed the IAT data strictly following the procedure suggested by Greenwald et al. [20]. That is, only Block 4 and Block 7 were included in the data analyses. Moreover, the first two trials of each block were excluded. Latencies “below 300 ms” (0.32% trials for analysis) and “above 3000 ms” (2.15% trials for analysis) were recorded to “300 ms” and “3000 ms” respectively. Four participants were excluded from the data analysis due to excessive error rates (above 30%). The average error rate of the remaining participants (*n* = 88) was 5.03%. As expected, participants did the “self-owned + positive vs. other-owned + negative” task (*M* = 821 ms, *SD* = 197 ms) faster than the “other-owned + positive vs. self-owned + negative” task (*M* = 949 ms, *SD* = 247 ms); *t* (87) = 6.85, *p* < .001, using log-transformed data. Therefore, our data suggests that the participants favored self-possessions more than other-possessions—a demonstration of the ownership effect.

Although we could subtract the log-transformed latency of the “self-owned + positive vs. other-owned + negative” task from that of the “other-owned + positive vs. self-owned + negative” task as a measure of the ownership effect, Greenwald, Nosek [25] suggested that D index, a variant of Cohen’s d, would be a more valid measure for the size of the IAT effect. Greenwald, Nosek [25] suggested to include Blocks 3, 4, 6, and 7 into data analysis. However, most participants had never participated in an IAT before, and they also needed to remember three items for self-owned possessions and three items for other-owned possessions, which led to relatively high error rates for Blocks 3 and 6 (the error rate for the practice block of “positive + self-owned vs. negative + other-owned” task, namely Block 3 for half of the participants and Block 6 for the other half, ranged from 0% to 59.1% with a mean of 4.65%, *SD* = 8.36%; the error rate for the practice block of the “positive + other-owned vs. negative + self-owned” task, namely Block 6 for half of the participants and Block 3 for the other half, ranged from 0% to 72.7% with a mean of 8.06%, *SD* = 9.72%). As a result, we only included Blocks 4 and 7 (the error rate for the test block of “positive + self-owned vs. negative + other-owned” task, namely Block 4 for half of the participants and Block 7 for the other half, ranged from 0% to 23.9% with a mean of 4.27%, *SD* = 4.87%; the error rate for the test block of the “positive + other-owned vs. negative + self-owned” task, namely Block 7 for half of the participants and Block 4 for the other half, ranged from 0% to 37.0% with a mean of 5.68%, *SD* = 5.90%) into data analysis. We first computed means and the *SDs* of correct latencies for each block and replaced each error latency with block mean plus two *SDs*. Then we calculated the difference between the mean response latencies for Blocks 4 and 7 and divided that difference by the pooled *SD* for all trials in Blocks 4 and 7. A larger D indicates a stronger ownership effect. One-sample t-test showed that the D was significantly larger than 0 (*M* = .30, *SD* = .46, *t*(87) = 6.13, *p* < .001), suggesting people prefer self-owned possessions to other-owned possessions when using a different algorithm.

#### The GNAT results

We analyzed the GNAT data using signal detection theory, in which *d*’ represented participants’ performance quality in each block [21]. The *d*’ was obtained by subtracting the false alarm rate from the hit rate after they were standardized with a probit function. Extreme cell values (0 or 1) were corrected following procedures from Greenwald and Banaji [26]. A paired t-test showed that participants did better when targets were self-possessions and positive words than when targets were self-possessions and negative words (*d*’_self-possessions & positive_ = 1.73, *SD* = .93 vs. *d*’_self-possessions & negative_ = 1.31, *SD* = 0.71; *t* (87) = 4.41, *p* < .001), consistent with the notion that participants held a positive attitude towards self-possessions. Participants also did better when targets were other-possessions and negative words than when targets were other-possessions and positive words (*d*’_other-possessions & negative_ = 1.47, *SD* = 0.84 vs. *d*’_other-possessions & positive_ = 0.91, *SD* = 0.75; *t* (87) = 6.42, *p* < .001), suggesting that participants held a negative attitude toward other-possessions.

Then the magnitude of self-enhancement was calculated by subtracting *d*’_self-possessions & negative_ from *d*’_self-possessions & positive_, while the magnitude of other-derogation was calculated by subtracting *d*’_other-possessions & positive_ from *d*’_other-possessions & negative_. The magnitude of self-enhancement was positively correlated with that of other-derogation (*r* = 0.349, *p* < .001). Moreover, paired t-test did not show significant difference between them (self-enhancement: *M* = 0.42, *SD* = 0.89, other-derogation: *M* = 0.56 *SD* = 0.82, *t*(87) = -1.35, *p* = .180), suggesting that the tendency of other-derogation is no less important than the tendency of self-enhancement. These findings provide primary evidence that both self-enhancement and other-derogation contribute to the ownership effect.

#### The correlations between IAT and GNAT

**Fig 1:**
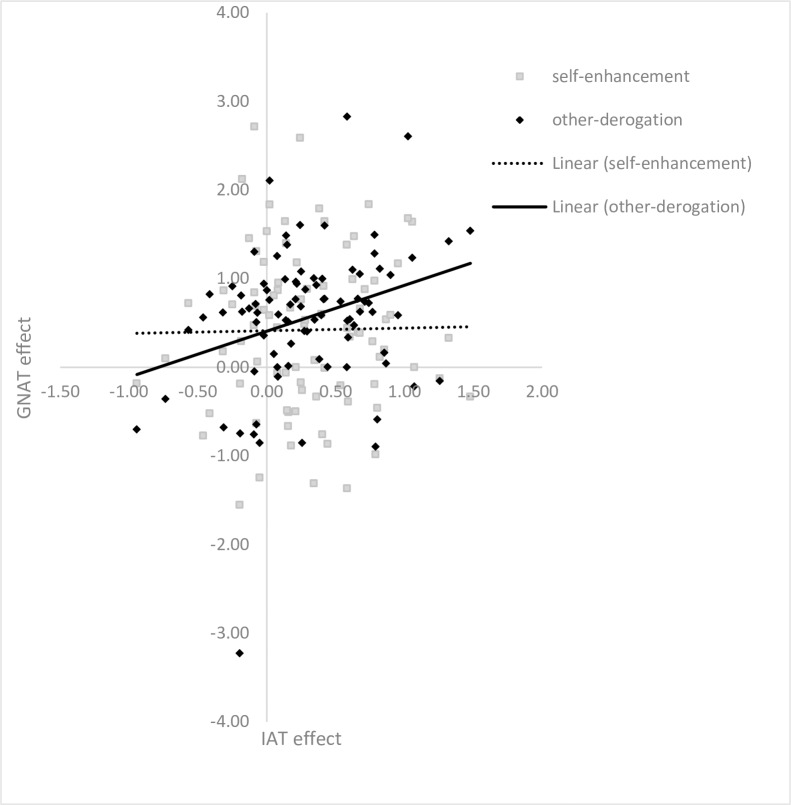
The Relationship between IAT and GNAT. The IAT effect = the D score in IAT; the GNAT effect for self-enhancement = *d*’_self-possessions & positive_−*d*’_self-possessions & negative_ in the GNAT; the GNAT effect for other-derogation = *d*’_other-possessions & negative_−*d*’_other-possessions & positive_ in the GNAT. Fitted lines were derived from linear regressions.

We investigated whether self-enhancement or other-derogation correlated with the ownership effect captured by the D index of IAT (see Fig 1). Correlational analysis showed that the ownership effect obtained from IAT was significantly correlated with other-derogation (*r* = 0.289, *p* = .006) but not with self-enhancement (*r* = 0.015, *p* = .888). Steiger's Z test showed that the difference between the two dependent correlations from a single sample was significant (z (85) = 2.25, *p* = .024)[27].

### Discussion

In study 1, we found support for the ownership effect through the IAT. In particular, we found that compared with other-owned possessions, participants held relatively stronger positive attitudes toward self-owned possessions. In addition, the results of GNAT suggest that participants engaged in both self-enhancement and other-derogation. Critically, however, only the other-derogation score derived from the GNAT task was associated with the IAT effects—we found a near zero correlation between GNAT self-enhancement score and the IAT ownership effect. Together, these findings suggest that the ownership effect demonstrated by IAT was mostly driven by the other-derogation, and confirmed the distinction between self-enhancement and other-derogation.

## Study 2

In Study 2, we asked participants to judge the ownership of different objects (self-possession or other-possession) which were preceded by either positive or negative adjectives [28] and recorded their brain potentials. Previous research has shown that in such affective priming, the incongruence between a prime (e.g., negative word or picture) and a target (e.g., positive word or picture) elicits increased evaluative incongruity, which can be indexed by increased late positive potentials (LPP) [23]. We hypothesized that if self-enhancement were taking place, then self-possessions paired with positive adjectives would result in evaluative congruity, and self-possessions paired with negative adjectives would result in evaluative incongruity. On the other hand, if other-derogation were taking place, other-possessions paired with negative adjectives would result in evaluative congruity, and other-possessions paired with positive adjectives would result in evaluative incongruity.

### Materials and Method

Thirty-two university students (14 females; *M*_*age*_ = 21.2, *SD* = 1.64) were recruited from universities in Beijing and paid 50 Chinese yuan (about $7.90). All the participants were right-handed and had normal or corrected-to-normal vision. The study was conducted in accordance with Declaration of Helsinki and was approved by the Institutional Review Board of School of Psychological and Cognitive Sciences Peking University. Written informed consent was obtained from all participants. The affective priming task used a 2 (ownership of the objects: self vs. other) × 2 (valence of the priming adjectives: positive vs. negative) within-participant design.

The task has been described in our past work (22). Participants were asked to imagine a scenario in which six objects (i.e., pen, candy, knapsack, cup, bread, and box) were assigned either to themselves or to an unspecified other person. Then the participants took part in an affective priming task [28] with EEG recordings. Each trial started with the presentation of a fixation cross at the center of the screen for 500 ms against a black background. Then a positive or negative adjective (white and *Song* font, size 32) that could be used to describe the quality of an object was presented for 800 ms. After a jittered interval of 200 ms, 300 ms, or 400 ms, one of the six memorized objects (e.g., *cup*) was presented for 1000 ms. This was followed by the presentation of two options, “self” and “other” (in words), randomly on the left or right side of the screen. The participants were asked to judge the ownership of the object by pressing a corresponding key as quickly as possible. The inter-trial interval (ITI) was 1000 ms.

The participant was seated about 1.5 m in front of a computer screen in a dimly lit and electromagnetically shielded room. The experiment was administered on a desktop computer with a 22-in. CRT display, using Presentation software (Neurobehavioral System Inc.) to control the presentation and timing of stimuli. Each participant received 2 blocks of 144 trials, with each of the four experimental conditions having 48 trials. In addition, there were 48 trials in which the objects were preceded by adjectives unrelated to the quality of the object and 48 trials in which the objects were not preceded by any words. These trials were used as fillers to control for possible response strategies. The 288 trials were randomized for each participant.

EEGs were recorded from 64 scalp sites using tin electrodes mounted in an elastic cap (Brain Products, Munich, Germany) according to the international 10–20 system. The vertical electrooculogram (VEOGs) was recorded supra-orbitally from the right eye. The horizontal EOG (HEOG) was recorded from an electrode placed at the outer canthus of the left eye. All EEGs and EOGs were referenced online to an external electrode which was placed on the tip of nose and were re-referenced offline to the mean of the left and right mastoids. All electrode impedance was kept below 5 kΩ. The bio-signals were amplified with a band pass from 0.016 to 100Hz and digitized on-line with a sampling frequency of 500 Hz.

EEG epochs of 1200 ms (with a 200-ms pre-stimulus baseline) were extracted offline for ERPs time-locked to the onset of the object names. Ocular artifacts were corrected with an eye-movement correction algorithm which employs a regression analysis in combination with artifact averaging [29]. Epochs were baseline-corrected by subtracting from each sample the average activity of that channel during the baseline period. All trials in which EEG voltages exceeded a threshold of ±80 μV during recording were excluded from further analysis. The EEG data were low-pass filtered below 30 Hz.

**Fig 2:**
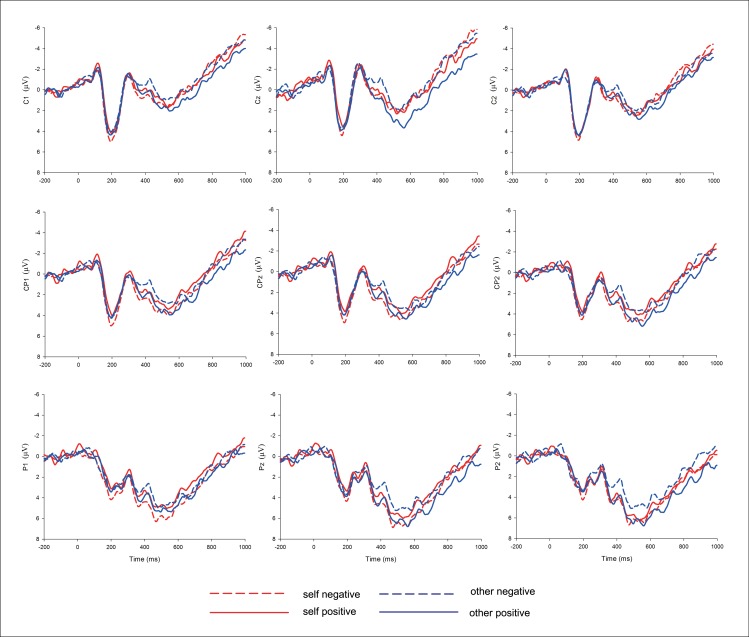
ERP Responses at the Nine Central-Posterior Electrodes, Time-Locked to the Onset of Object Nouns.

Based on visual inspection of the ERP waveforms (see Fig 2), we selected the ERP responses in the 350–800 ms (late positive potential; LPP) time window for statistical analysis. The Greenhouse-Geisser correction for violation of the assumption of sphericity was applied where appropriate. The Bonferroni correction was used for multiple comparisons. We focused mainly on LPP, a neural signal associated with evaluative incongruity [30–32].

### Results

There were five participants who displayed excessive artifacts in EEG recording. These participants were excluded from further analysis. We did not follow the classic procedure of the affective priming task [28]. The standard affective priming task requires participant to react to the stimuli (i.e., the objects in the present study) as soon as they see them and record the reaction time. However, in the present study, participants saw the stimuli for 1000ms, which enabled us to record the EEG signal, before they could react to them. As a result, response time data was not very informative in inferring evaluative incongruity and were not analyzed here [22].

We examined the results of the affective priming task to evaluate whether self-enhancement or other-derogation was taking place. For LPP, mean amplitudes in the time window of 350–800 ms averaged across 9 central-posterior electrodes (i.e., C1, Cz, C2, CP1, CPz, CP2, P1, Pz, P2; see Fig 2) where this effect appeared largest were computed for analysis [22, 33, 34]. A 2 × 2 repeated ANOVA found significant interaction between ownership and valence, *F*(1,26) = 10.29, *p* = .004. A simple-effect test revealed that positive-framed other-possessions (2.96 μV) elicited more positive-going LPP than negative-framed other-possessions (1.97 μV), *F*(1, 26) = 5.83, *p* = .023. The results suggest that other-possessions are more closely linked with negative valence. Framing these objects as positive evoked significant evaluative incongruence relative to those framed as negative, suggesting that people devaluate other-possessions. However, the LPP difference for self-possessions was in the hypothesized direction and did not reach statistical significance (positive-framed self-possessions vs. negative-framed self-possessions, 2.23 μV vs. 2.70 μV), *F*(1, 26) = 1.86, *p* = .18. Neither the main effect of ownership nor valence was significant, *p* = .99, and *p* = .39, respectively.

### Discussion

Adopting the EEG technique, Study 2 found a significant difference of LPP between positive-framed vs. negative-framed other possessions, while the difference of LPP between positive-framed vs. negative-framed self-owned possessions did not reach significance. These results suggest that self-enhancement is relatively less salient compared to other-derogation, a finding that is consistent with the IAT result in Study 1.

## General Discussions

Overall, the findings from these two studies suggest that the ownership effect can be driven by other-derogation. Study 1 used the GNAT and found people associated other-possessions more with negative attributes than positive attributes. Study 2 adopted ERP technology and found positive-framed other-owned objects elicited significant evaluative incongruity relative to negative-framed other-owned objects, and this difference was indexed by increased LPP, a neural signal associated with incongruence evaluation. These findings help us to better understand the underlying mechanism of the ownership effect.

Although self-enhancement was demonstrated in the GNAT in Study 1, it did not predict the ownership effects measured by the IAT. Moreover, in Study 2, self-enhancement indexed by ERP signals failed to reach significance. While the lack of significance could well be due to a sample size problem, the data suggested that the ownership effect examined in the present research seemed to be driven by other-derogation but not self-enhancement, which was inconsistent with previous literature which mainly focused on self-enhancement. For example, Dommer and Swaminathan [6] found that self-threat led to stronger self-enhancement but not stronger other-derogation in the ownership effect. However, this inconsistency is likely to result from the different paradigms. For instance, in Dommer and Swaminathan [6]'s research, participants were assigned to the role of either owner or non-owner. Ownership was treated as a between-subjects variable. In contrast, in the present research, participants had to differentiate self-possessions and other-possessions directly; thus they had to compare self-possessions and other-possessions directly. It is possible that the ownership effect with comparison and without comparison may work on separate principles. Future work should investigate these issues further.

In addition, participants might find it odd to be asked to compare self-possessions versus other-possessions that are identical or similar (for the purpose of showing the ownership effect). Moreover, participants might want to be rational in their explicit judgment or to try to avoid being arrogant [16], making it difficult to observe the ownership effect in a paradigm involving direct comparison. These are the reasons that most previous research tended to assign participants to different roles (owner vs. the non-owner) rather than asking the same participants to make comparisons between self- and other-possessions. Therefore, we did not adopt explicit measures which are likely to suffer from the self-representative bias. Future research could benefit by developing better explicit measures which are not susceptible to the self-representative bias. Our research utilized two different approaches to examine the ownership effect with direct comparison, ruling out bias from social desirability and the need to answer questions in a reasonable way [16]. These methodologies will also allow us to disentangle differences between the different types of ownership effects in future studies.

From the perspective of methodology, the present research shows that GNAT is able to capture the implicit ownership effect shown by the IAT [19]. In addition, the IAT effect in the present research seems to be driven more by the negative association (i.e., other-derogation) compared with the positive association (i.e., self-enhancement). This leaves unanswered questions for the widely adopted paradigm of IAT in general. That is, which direction drives the IAT effects: negative or positive associations? And which factors could influence their relative salience?

Some limitations have to be noted. First, the implicit measures have well-known confounds of their own. As “salience asymmetry” [35] has suggested, the observed strong association between negative words and other-owned products may be driven by having similar level of salience rather than shared associative meaning. This alternative explanation works for both IAT and GNAT. Second, the self-enhancement/other-derogation measured by GNAT did not exclusively focus on the self/other because in the blocks measuring self-enhancement, other-owned possessions were still shown. An improved way might be measuring self-enhancement/other-derogation while not involving other-owned/self-owned possessions as noise.

Moreover, objects were described as owned by the self or other through imagined scenarios. Previous research has shown that imagination is sufficient to have an effect on perceived ownership [36]. However, there is still relatively little evidence showing that ownership caused by imagination and by fact are identical. It is possible that actually owning an object makes the self-enhancement stronger than simply imagination, which can explain why self-enhancement seems less salient than other-derogation. This possibility should be explored in future research.

Another possible explanation is that our studies were conducted in East Asia. Cross-cultural research has demonstrated that people in East Asian and Western cultures differ in their emphasis of the self versus others [37]. While people in the West tend to focus on personal achievement and individual benefit, people from Eastern cultures are more likely to evaluate themselves in relation to others [38]. This emphasis on the self within Western cultures may lead to self-enhancement’s being the primary mechanism of the ownership effect within Western cultures—a finding in stark juxtaposition to our results with Chinese participants. Future replications of our studies with Western samples could shed light on this interesting possibility.

Moreover, our proposition that other-derogation is not mere lack of self-enhancement is in line with the body ownership literature where it is speculated that the sense of disownership experienced by patients towards their body parts cannot be reduced to the mere lack of ownership [39]. Therefore, by drawing on the ERP and electrophysiology studies on hand ownership [40–42], future research could further inform the comparison between self-enhancement and other-derogation, and contextualize the ERP results in the present research.

## Supporting Information

S1 File Data of Study 1. (PDF)

S2 File Data of Study 2. (PDF)
