# Supplementary material for: Ownership Effect Can Be a Result of Other-Derogation: Evidence from Behavioral and Electrophysiological Studies
Source: PLoS One. 2016 Nov 4;11(11):e0166054. doi: 10.1371/journal.pone.0166054 (PMC5096666; doi:10.1371/journal.pone.0166054)
Supplement: S1 File — (PDF) [file pone.0166054.s001.pdf]

subj	collect	ORDER	ERRORPC	valid	LATENCY1	LATENCY2	LN_LAT1	LN_LAT2
1702	2	2	0.694444	1	1072.0217	1124.1739	6.7961	6.888865
1633	2	1	2.777778	1	1629.6522	893.3913	7.259572	6.724461
1615	2	1	12.5	1	1253.3171	1232.3415	6.981556	6.928897
1630	2	2	0.694444	1	883.78261	909.82609	6.686063	6.701158
2002	2	2	0.694444	1	963.04348	978.68889	6.794323	6.790679
1642	2	2	0.694444	1	844.5	803.52174	6.604992	6.587224
1733	2	1	0.694444	1	747.44444	547.08696	6.570508	6.294925
1603	2	1	1.388889	1	630.95652	740.6	6.420787	6.574348
1652	2	2	1.388889	1	587.17778	694.5	6.347447	6.499852
34	1	2	1.388889	1	667.84444	728.11111	6.489907	6.536518
1645	2	1	1.388889	1	664.5	671	6.452148	6.453997
1730	2	2	1.388889	1	1211.8261	1051.7727	6.980172	6.806469
1641	2	1	1.388889	1	890.70455	880.58696	6.728257	6.686918
23	1	1	1.388889	1	742.13044	665.82222	6.579478	6.465888
1650	2	2	1.388889	1	1323.2889	1012.4783	7.02586	6.829888
1619	2	1	1.388889	1	1184.0889	928.65217	7.012615	6.760786
1627	2	1	1.388889	1	1067.5435	711.8	6.871944	6.466455
1737	2	1	2.083333	1	807.58696	775.53488	6.63451	6.596467
1634	2	2	2.083333	1	662.28261	681.46667	6.443768	6.491232
1601	2	1	2.083333	1	905.09302	996.15217	6.690671	6.759722
2001	2	1	2.083333	1	779.6	764.84444	6.623651	6.568787
36	1	2	2.083333	1	943.57778	920.91111	6.770276	6.787769
1639	2	1	2.083333	1	639.51111	595.86364	6.435761	6.354539
1616	2	2	2.083333	1	706.66667	642.93333	6.533598	6.416793
25	1	1	2.083333	1	1262.9111	850.08889	7.075824	6.693144
39	1	1	2.083333	1	764.88636	659.88889	6.610644	6.46985
1660	2	2	2.083333	1	1097	727.3913	6.929729	6.507418
1640	2	2	2.083333	1	1126.8409	650.65217	6.937311	6.441509
1713	2	1	2.777778	1	822.63044	758.7907	6.654787	6.536925
21	1	1	2.777778	1	706.21739	712.75	6.541347	6.535757
1718	2	2	2.777778	1	779.97778	754.59091	6.627448	6.560642
1724	2	2	2.777778	1	984.64444	805.71111	6.847531	6.651912
1728	2	2	2.777778	1	1346.75	1348.9348	7.057232	7.059865
28	1	2	2.777778	1	654.13636	585.80435	6.464029	6.366796
1723	2	1	2.777778	1	967.45455	888.97826	6.786027	6.700109
1624	2	2	2.777778	1	991.86957	841.66667	6.768857	6.670492
1738	2	2	2.777778	1	896.04651	790.13044	6.677129	6.583408
1714	2	2	2.777778	1	873.17778	699.37778	6.665512	6.504196
1703	2	1	2.777778	1	729.65909	609.72727	6.555898	6.392323
1725	2	1	2.777778	1	776.86364	650.15217	6.613309	6.456052
35	1	1	2.777778	1	1309.1591	787.52174	7.114303	6.6182
1610	2	2	3.472222	1	890.04444	913.73333	6.654409	6.679199
1	2	1	3.472222	1	696.41304	563.07143	6.473316	6.314942
26	1	2	3.472222	1	538.63636	498.51111	6.277738	6.194982
30	1	2	3.472222	1	649.5	575.06667	6.453729	6.341343
1644	2	2	3.472222	1	972.39535	754.34783	6.796212	6.565854
1606	2	2	4.166667	1	991.28571	1108.1818	6.776251	6.847061
24	1	2	4.166667	1	924.17778	1026.8222	6.754915	6.878054
1701	2	1	4.166667	1	844.32558	726.22727	6.614645	6.508476
1744	2	2	4.166667	1	1164.25	940.22727	6.990034	6.761175
1617	2	1	4.166667	1	1021.3261	781.07143	6.815415	6.600558
1715	2	1	4.166667	1	1263.878	1029.5217	6.968811	6.799532

1605	2	1	4.166667	1	1287.093	821.32609	7.103711	6.656801
1736	2	2	4.861111	1	888.37778	891.75556	6.728952	6.731008
1721	2	1	4.861111	1	1023.4419	907.52273	6.806197	6.648141
1726	2	2	4.861111	1	918.44186	891.11111	6.767028	6.732914
1646	2	2	4.861111	1	1328.6744	1092.3333	7.044413	6.865595
1712	2	2	4.861111	1	696.65116	579.4	6.499577	6.334463
1648	2	2	5.555556	1	681.69048	642.88095	6.47169	6.424757
1069	2	1	5.555556	1	825.38095	640.6	6.627827	6.420477
1731	2	1	6.25	1	1024.5	1319.7805	6.808955	6.970237
33	1	1	6.25	1	773.04651	775.90476	6.621251	6.601715
27	1	1	6.25	1	900.15556	842.69048	6.759847	6.63574
1708	2	2	6.25	1	848.11628	749.24444	6.650163	6.52305
1705	2	1	6.25	1	686.925	591.06977	6.499285	6.353094
31	1	1	6.25	1	1228.5814	836.51111	7.028107	6.668224
2007	2	1	6.944444	1	806.97674	736.375	6.631072	6.54533
1707	2	1	6.944444	1	1233.75	662.90909	7.073079	6.456689
1638	2	2	7.638889	1	945.69048	708.30233	6.74892	6.509511
2005	2	1	7.638889	1	1163.119	735.31818	6.991467	6.564061
1716	2	2	8.333333	1	586.60976	543.63415	6.338111	6.263652
32	1	2	8.333333	1	1421.4884	967.07143	7.147637	6.849088
1643	2	1	8.333333	1	1458.7857	797.3913	7.124324	6.59406
1729	2	1	9.027778	1	990	922.53333	6.79269	6.706496
1635	2	1	9.722222	1	1046.625	781.13636	6.840006	6.581109
38	1	2	11.111111	1	1333.4222	1545.5833	7.107986	7.190253
1614	2	2	11.111111	1	631.3	719.90476	6.410742	6.531897
55	2	1	11.111111	1	1261.7949	1235.8444	7.03052	7.011919
1655	2	1	11.111111	1	1016.6829	595.16667	6.829182	6.353871
1717	2	1	11.805556	1	922.05	733.97436	6.721272	6.495379
1704	2	2	11.805556	1	1062.8205	1056.5581	6.798615	6.833502
1618	2	2	12.5	1	801.19048	912	6.618903	6.722079
56	2	2	14.583333	1	997.38462	968.23077	6.828327	6.752233
1735	2	1	20.138889	1	931.41177	760.56098	6.766935	6.587309
1740	2	2	22.916667	1	765.10256	900.11429	6.578337	6.704877
2003	2	1	32.638889	0	1691	1096.5938	7.357106	6.930913
37	1	1	4.166667	1	1689.5476	1089.1739	7.311833	6.864388
40	1	2	11.805556	1	743.02381	565.35897	6.581144	6.27623
1719	2	1	40.972222	0	792.72222	726.90476	6.634503	6.535814
1620	2	2	28.472222	1	754.37931	708.74419	6.326161	6.513808
1661	2	1	32.638889	0	300	1287.4872	5.703782	7.090523
1628	2	2	35.643564	0	300	418.29167	5.703782	5.910921

D2SD_T	NTRIAL_P	NTRIAL_T	ERROR1_F	ERROR2_F	ERROR1_1	ERROR2_1	ERROR2_1	ERROR2_2
0.051716	48	95	4.545455	0	0	0	2.272727	0
-0.768353	47	96	18.181818	0	0	0	9.090909	0
-0.080559	48	95	27.272727	9.090909	10.869565	10.869565	19.071146	9.980237
0.127305	48	96	0	4.545455	0	0	0	2.272727
-0.019877	48	96	0	0	0	2.173913	0	1.086957
-0.075243	48	96	4.545455	0	0	0	2.272727	0
-0.802962	48	96	0	0	2.173913	0	1.086957	0
0.568199	48	96	4.545455	0	0	2.173913	2.272727	1.086957
0.460307	48	95	4.545455	0	2.173913	0	3.359684	0
0.311937	48	96	0	0	2.173913	2.173913	1.086957	1.086957
0.075211	48	96	4.545455	4.545455	0	0	2.272727	2.272727
-0.133722	48	96	0	0	0	4.347826	0	2.173913
-0.156409	48	96	0	0	4.347826	0	2.173913	0
-0.293949	48	96	0	4.545455	0	2.173913	0	3.359684
-0.341864	48	96	4.545455	0	2.173913	0	3.359684	0
-0.584325	48	96	4.545455	0	2.173913	0	3.359684	0
-0.603173	48	96	4.545455	0	0	2.173913	2.272727	1.086957
0.19559	48	96	0	0	0	6.521739	0	3.26087
0.191895	48	96	4.545455	4.545455	0	2.173913	2.272727	3.359684
0.089391	48	95	0	0	6.521739	0	3.26087	0
0.020766	48	96	4.545455	0	2.173913	2.173913	3.359684	1.086957
-0.078366	48	96	4.545455	0	2.173913	2.173913	3.359684	1.086957
-0.211361	48	96	0	0	2.173913	4.347826	1.086957	2.173913
-0.218939	48	96	4.545455	0	2.173913	2.173913	3.359684	1.086957
-0.590551	48	96	4.545455	0	2.173913	2.173913	3.359684	1.086957
-0.623256	48	96	0	0	4.347826	2.173913	2.173913	1.086957
-0.741969	48	96	13.636364	0	0	0	6.818182	0
-1.057829	48	96	0	4.545455	4.347826	0	2.173913	2.272727
0.088962	48	96	4.545455	0	0	6.521739	2.272727	3.26087
0.074729	48	96	4.545455	4.545455	0	4.347826	2.272727	4.44664
-0.021101	48	96	4.545455	0	2.173913	4.347826	3.359684	2.173913
-0.084698	48	96	4.545455	4.545455	2.173913	2.173913	3.359684	3.359684
-0.210675	48	95	4.545455	4.545455	4.347826	0	4.44664	2.272727
-0.243731	48	96	9.090909	0	4.347826	0	6.719368	0
-0.249962	48	96	9.090909	0	4.347826	0	6.719368	0
-0.281429	48	96	13.636364	0	0	2.173913	6.818182	1.086957
-0.40397	48	96	4.545455	0	6.521739	0	5.533597	0
-0.414054	48	96	4.545455	4.545455	2.173913	2.173913	3.359684	3.359684
-0.63461	48	96	0	0	4.347826	4.347826	2.173913	2.173913
-0.712395	48	96	4.545455	4.545455	4.347826	0	4.44664	2.272727
-1.02594	48	96	9.090909	0	4.347826	0	6.719368	0
0.020547	48	96	9.090909	4.545455	2.173913	2.173913	5.632411	3.359684
-0.247509	48	95	4.545455	0	0	8.695652	2.272727	4.347826
-0.420818	46	94	9.090909	0	4.347826	2.173913	6.719368	1.086957
-0.581368	48	96	4.545455	4.545455	4.347826	2.173913	4.44664	3.359684
-0.661582	48	96	0	4.545455	6.521739	0	3.26087	2.272727
0.248924	48	96	0	0	8.695652	4.347826	4.347826	2.173913
0.177705	48	96	4.545455	9.090909	2.173913	2.173913	3.359684	5.632411
-0.348173	48	96	4.545455	0	6.521739	4.347826	5.533597	2.173913
-0.38249	48	96	4.545455	4.545455	4.347826	4.347826	4.44664	4.44664
-0.396589	48	96	0	9.090909	0	8.695652	0	8.893281
-0.586501	48	96	4.545455	0	10.869565	0	7.70751	0

-0.954027	48	96	13.636364	0	6.521739	0	10.079051	0
0.066687	48	96	22.727273	0	2.173913	2.173913	12.450593	1.086957
-0.001093	48	96	4.545455	4.545455	6.521739	4.347826	5.533597	4.44664
-0.051586	48	96	9.090909	4.545455	6.521739	2.173913	7.806324	3.359684
-0.442166	48	96	13.636364	0	6.521739	2.173913	10.079051	1.086957
-0.536947	48	96	13.636364	0	6.521739	2.173913	10.079051	1.086957
-0.172661	48	93	0	0	8.695652	8.695652	4.347826	4.347826
-0.678417	48	95	4.545455	9.090909	8.695652	2.173913	6.620553	5.632411
0.316965	48	96	0	4.545455	4.347826	10.869565	2.173913	7.70751
0.093562	48	96	9.090909	0	6.521739	8.695652	7.806324	4.347826
0.079572	48	96	9.090909	9.090909	2.173913	8.695652	5.632411	8.893281
-0.360843	47	96	13.636364	9.090909	6.521739	2.173913	10.079051	5.632411
-0.820842	48	94	0	0	13.043478	6.521739	6.521739	3.26087
-0.900147	48	96	13.636364	4.545455	6.521739	2.173913	10.079051	3.359684
-0.14207	48	96	4.545455	0	6.521739	13.043478	5.533597	6.521739
-1.322563	48	96	13.636364	4.545455	4.347826	4.347826	8.992095	4.44664
-0.676221	48	96	13.636364	4.545455	8.695652	6.521739	11.166008	5.533597
-1.256917	48	96	18.181818	0	8.695652	4.347826	13.438735	2.173913
-0.158079	48	90	4.545455	4.545455	10.869565	10.869565	7.70751	7.70751
-0.867757	48	96	0	22.727273	6.521739	8.695652	3.26087	15.711462
-1.071503	48	96	18.181818	18.181818	8.695652	0	13.438735	9.090909
-0.273648	48	96	13.636364	9.090909	10.869565	2.173913	12.252964	5.632411
-0.78355	48	96	18.181818	9.090909	13.043478	4.347826	15.612648	6.719368
0.94285	48	96	0	13.636364	2.173913	21.73913	1.086957	17.687747
0.413188	48	96	18.181818	4.545455	13.043478	8.695652	15.612648	6.620553
-0.258154	48	96	27.272727	4.545455	15.217391	2.173913	21.245059	3.359684
-0.854413	48	93	18.181818	13.636364	10.869565	8.695652	14.525692	11.166008
-0.138863	48	93	13.636364	4.545455	13.043478	15.217391	13.339921	9.881423
-0.148801	48	96	4.545455	18.181818	15.217391	6.521739	9.881423	12.351779
0.185643	48	95	9.090909	27.272727	8.695652	8.695652	8.893281	17.98419
-0.177641	48	96	18.181818	4.545455	15.217391	15.217391	16.699605	9.881423
-0.790535	48	96	22.727273	27.272727	26.086957	10.869565	24.407115	19.071146
0.734551	48	96	9.090909	59.090909	15.217391	23.913043	12.15415	41.501976
-1.106122	48	95	40.909091	18.181818	36.956522	30.434783	38.932806	24.3083
-0.783752	48	95	9.090909	0	8.695652	0	8.893281	0
-0.42081	48	92	13.636364	9.090909	8.695652	15.217391	11.166008	12.15415
0.706645	25	91	27.272727	59.090909	21.73913	54.347826	24.505929	56.719368
-1.47891	32	67	72.727273	13.636364	36.956522	6.521739	54.841897	10.079051
#NULL!	31	49	36.363636	45.454545	43.478261	15.217391	39.920949	30.335968
-0.228843	27	10	40.909091	4.545455	66.666667	47.826087	53.787879	26.185771

AVELTNCY	NTRIALS	mineg600hi	mineg600fa	mineg600m	mineg600c	mineb600hi	mineb600fa	mineb600m
1486.8889	144	16	11	14	19	17	2	13
1785.6181	144	19	7	11	23	18	9	12
1597.8681	144	25	9	5	21	15	8	15
1068.3958	144	23	7	7	23	20	20	10
1079.9583	144	26	11	4	19	21	11	9
955.92361	144	29.65	9	0.35	21	28	6	2
803.4375	144	23	8	7	22	25	6	5
724.79861	144	29	3	1	27	26	3	4
651.04167	144	23	8	7	22	24	3	6
705.625	144	29	3	1	27	25	3	5
695.99306	144	27	10	3	20	28	6	2
1315.5833	144	26	0.35	4	29.65	22	4	8
983.59722	144	23	11	7	19	22	4	8
779.42361	144	26	6	4	24	23	11	7
1357.1111	144	24	9	6	21	24	1	6
1252.7153	144	26	9	4	21	28	2	2
954.24306	144	28	5	2	25	28	8	2
1034.0833	144	19	16	11	14	25	6	5
708.72917	144	27	11	3	19	25	6	5
1034.3333	144	24	3	6	27	15	3	15
818.88194	144	24	5	6	25	22	15	8
1063.2569	144	19	7	11	23	17	5	13
687.71528	144	25	5	5	25	25	5	5
742.95139	144	26	5	4	25	17	7	13
1313.9861	144	24	4	6	26	24	2	6
734.22222	144	28	2	2	28	23	3	7
1050.5208	144	29	3	1	27	26	13	4
1183.4583	144	29	2	1	28	25	7	5
810.30556	144	29.65	2	0.35	28	22	10	8
790.05556	144	28	2	2	28	25	7	5
827.17361	144	29	3	1	27	15	3	15
1044.0208	144	25	11	5	19	13	9	17
1599.1181	144	26	24	4	6	29.65	28	0.35
673.79861	144	29.65	2	0.35	28	24	11	6
1097.7778	144	26	10	4	20	18	9	12
958.05556	144	28	2	2	28	28	5	2
894.51389	144	16	5	14	25	25	6	5
857.89583	144	27	11	3	19	22	14	8
706.19444	144	28	4	2	26	17	5	13
757.20833	144	29.65	6	0.35	24	28	7	2
1181.4722	144	29	8	1	22	18	9	12
931.125	144	29	9	1	21	27	10	3
686.58333	144	27	13	3	17	27	11	3
534.15278	144	29.65	2	0.35	28	24	3	6
670.89583	144	27	1	3	29	22	4	8
1070.4653	144	26	10	4	20	22	9	8
1187.5	144	25	12	5	18	20	14	10
1031.125	144	16	2	14	28	8	14	22
806.15278	144	28	13	2	17	25	8	5
1232.7083	144	29	7	1	23	19	10	11
1092.5208	144	26	6	4	24	22	7	8
1173.6528	144	23	5	7	25	21	7	9

1312.1319	144	28	8	2	22	21	10	9
1007.5139	144	24	6	6	24	27	11	3
1043.5972	144	27	12	3	18	20	20	10
1068.6042	144	29	12	1	18	26	13	4
1295.8611	144	20	21	10	9	19	10	11
669.13889	144	27	14	3	16	24	7	6
656.27083	144	27	6	3	24	26	11	4
861.59722	144	27	5	3	25	25	8	5
1330.1528	144	20	7	10	23	23	12	7
816.88889	144	27	11	3	19	21	8	9
912.29167	144	26	7	4	23	21	8	9
1000.0208	144	26	2	4	28	29	4	1
661.50694	144	28	8	2	22	27	7	3
1062.1667	144	25	6	5	24	18	5	12
806.16667	144	25	7	5	23	23	20	7
1055.1736	144	23	5	7	25	26	12	4
992.8125	144	22	7	8	23	22	11	8
987.94444	144	18	6	12	24	18	5	12
608.50694	144	20	5	10	25	27	8	3
1413.2708	144	14	10	16	20	8	10	22
1314.3333	144	25	10	5	20	20	5	10
1225.3403	144	26	7	4	23	22	7	8
955.53472	144	24	10	6	20	24	8	6
1737.3125	144	9	11	21	19	20	20	10
766.96528	144	23	11	7	19	22	5	8
1458.8958	144	19	14	11	16	18	10	12
852.04861	144	13	8	17	22	14	11	16
947.97222	144	15	8	15	22	20	12	10
1147.3958	144	21	4	9	26	24	3	6
916.30556	144	23	10	7	20	21	11	9
1163.2639	144	16	10	14	20	20	5	10
1144.1875	144	19	13	11	17	21	5	9
879.48611	144	21	16	9	14	19	15	11
1516.2431	144	9	4	21	26	13	9	17
1737.7708	144	28	8	2	22	22	9	8
729.22222	144	22	13	8	17	25	17	5
749.21528	144	29.65	29	0.35	1	21	18	9
771.29167	144	28	29	2	1	29.65	29.65	0.35
730.72222	144	0.35	0.35	0.35	0.35	0.35	0.35	0.35
650.81188	101	#NULL!	#NULL!	#NULL!	#NULL!	#NULL!	#NULL!	#NULL!

mineb600co	hisg600hit	hisg600fal	hisg600mis	hisg600cor	hisb600hit	hisb600fal	hisb600mis	hisb600cor
28	14	4	16	26	13	11	17	19
21	15	5	15	25	22	5	8	25
22	19	22	11	8	26	28	4	2
10	27	28	3	2	25	21	5	9
19	18	14	12	16	18	6	12	24
24	26	21	4	9	28	11	2	19
24	28	8	2	22	27	12	3	18
27	24	5	6	25	28	7	2	23
27	17	4	13	26	26	7	4	23
27	27	5	3	25	24	7	6	23
24	28	7	2	23	28	14	2	16
26	24	5	6	25	25	1	5	29
26	16	10	14	20	14	4	16	26
19	19	8	11	22	21	6	9	24
29	24	8	6	22	25	2	5	28
28	19	13	11	17	29	2	1	28
22	26	12	4	18	27	8	3	22
24	29	4	1	26	8	11	22	19
24	27	8	3	22	23	10	7	20
27	22	5	8	25	20	4	10	26
15	17	8	13	22	26	8	4	22
25	19	2	11	28	19	2	11	28
25	23	10	7	20	24	3	6	27
23	24	13	6	17	26	6	4	24
28	13	9	17	21	17	9	13	21
27	24	6	6	24	28	3	2	27
17	23	18	7	12	27	16	3	14
23	23	10	7	20	26	3	4	27
20	25	11	5	19	28	4	2	26
23	23	17	7	13	23	11	7	19
27	18	12	12	18	28	4	2	26
21	26	19	4	11	24	9	6	21
2	28	29.65	2	0.35	25	25	5	5
19	19	9	11	21	28	5	2	25
21	15	7	15	23	25	6	5	24
25	28	8	2	22	28	2	2	28
24	18	10	12	20	24	6	6	24
16	22	18	8	12	25	13	5	17
25	17	11	13	19	23	12	7	18
23	20	14	10	16	23	9	7	21
21	9	13	21	17	25	3	5	27
20	24	11	6	19	26	10	4	20
19	27	18	3	12	27	10	3	20
27	28	7	2	23	28	2	2	28
26	27	2	3	28	28	3	2	27
21	18	12	12	18	17	4	13	26
16	25	19	5	11	20	4	10	26
16	5	5	25	25	11	5	19	25
22	25	10	5	20	25	5	5	25
20	19	9	11	21	20	9	10	21
23	24	9	6	21	26	6	4	24
23	15	4	15	26	21	4	9	26

20	21	6	9	24	26	6	4	24
19	21	19	9	11	25	17	5	13
10	18	20	12	10	21	13	9	17
17	23	10	7	20	19	5	11	25
20	18	17	12	13	19	18	11	12
23	22	18	8	12	26	15	4	15
19	26	9	4	21	28	6	2	24
22	26	11	4	19	25	4	5	26
18	20	16	10	14	19	8	11	22
22	27	7	3	23	21	7	9	23
22	23	12	7	18	25	7	5	23
26	21	4	9	26	27	3	3	27
23	29	15	1	15	29	4	1	26
25	19	6	11	24	26	4	4	26
10	17	16	13	14	24	7	6	23
18	18	16	12	14	25	8	5	22
19	17	15	13	15	18	5	12	25
25	25	13	5	17	23	12	7	18
22	28	13	2	17	24	6	6	24
20	11	7	19	23	20	15	10	15
25	25	10	5	20	24	11	6	19
23	19	6	11	24	25	8	5	22
22	20	15	10	15	27	10	3	20
10	22	20	8	10	13	19	17	11
25	23	14	7	16	21	4	9	26
20	26	19	4	11	16	17	14	13
19	16	12	14	18	11	6	19	24
18	17	13	13	17	21	11	9	19
27	14	7	16	23	21	2	9	28
19	15	9	15	21	13	2	17	28
25	19	7	11	23	13	2	17	28
25	20	13	10	17	20	23	10	7
15	21	17	9	13	19	19	11	11
21	9	14	21	16	13	6	17	24
21	16	10	14	20	27	7	3	23
13	15	14	15	16	24	6	6	24
12	21	20	9	10	25	21	5	9
0.35	27	29.65	3	0.35	29	27	1	3
0.35	0.35	0.35	0.35	0.35	1	2	29	28
#NULL!	#NULL!	#NULL!	#NULL!	#NULL!	#NULL!	#NULL!	#NULL!	#NULL!

dmineg600	dmineb600	dhisg600	dhisb600	gnatmine	gnathis	IATeffect
0.424347	1.66898	1.02712	0.172801	-1.244633	-0.854319	0.051716
1.068608	0.777748	0.967422	1.590347	0.290861	0.622926	-0.768353
1.491822	0.622926	-0.282231	-0.390314	0.868896	-0.108083	-0.080559
1.455827	0	-0.219534	0.443021	1.455827	0.662555	0.127305
1.451466	0.865095	0.336999	1.094968	0.586371	0.757969	-0.019877
2.792333	2.342707	0.586371	1.841781	0.449626	1.25541	-0.075243
1.350839	1.809043	2.124012	1.534899	-0.458204	-0.589113	-0.802962
3.115466	2.392323	1.809043	2.228999	0.723143	0.419956	0.568199
1.350839	2.123173	1.278666	1.838685	-0.772334	0.560019	0.460307
3.115466	2.248973	2.248973	1.569535	0.866493	-0.679439	0.311937
1.712279	2.342707	2.228999	1.584738	-0.630428	-0.644262	0.075211
3.378704	1.733697	1.809043	2.801336	1.645007	0.992293	-0.133722
1.068608	1.733697	0.514379	1.02712	-0.665089	0.512741	-0.156409
1.952393	1.068608	0.963621	1.366022	0.883785	0.402401	-0.293949
1.366022	2.675536	1.464547	2.468508	-1.309514	1.003961	-0.341864
1.635172	3.002172	0.508589	3.335001	-1.367	2.826412	-0.584325
2.468508	2.124012	1.364119	1.904477	0.344496	0.540359	-0.603173
0.257043	1.809043	2.944686	-0.282231	-1.552	-3.226917	0.19559
1.622246	1.809043	1.904477	1.158641	-0.186796	-0.745837	0.191895
2.123173	1.281552	1.590347	1.541499	0.841621	-0.048848	0.089391
1.809043	0.622926	0.79082	1.733697	1.186117	0.942878	0.020766
1.068608	1.135316	1.841781	1.841781	-0.066707	0	-0.078366
1.934843	1.934843	1.158641	2.123173	0	0.964532	-0.211361
2.078193	0.895807	1.009515	1.952393	1.182386	0.942878	-0.218939
1.952393	2.342707	0.356507	0.692295	-0.390314	0.335788	-0.590551
3.002172	2.009465	1.683242	2.782638	0.992707	1.099395	-0.623256
3.115466	1.278666	0.474566	1.1979	1.836801	0.723334	-0.741969
3.335001	1.695335	1.158641	2.392323	1.639666	1.233683	-1.057829
3.769018	1.053653	1.308116	2.611858	2.715365	1.303741	0.088962
3.002172	1.695335	0.560019	1.068608	1.306837	0.508589	0.074729
3.115466	1.281552	0.506694	2.611858	1.833915	2.105163	-0.021101
1.308116	0.356507	0.770077	1.366022	0.95161	0.595945	-0.084698
0.26915	0.766846	-0.766846	0	-0.497696	0.766846	-0.210675
3.769018	1.182316	0.865095	2.468508	2.586702	1.603412	-0.243731
1.541499	0.777748	0.727913	1.809043	0.763751	1.08113	-0.249962
3.002172	2.468508	2.124012	3.002172	0.533664	0.87816	-0.281429
1.051073	1.809043	0.684074	1.683242	-0.757969	0.999168	-0.40397
1.622246	0.706577	0.369579	1.135316	0.915669	0.765737	-0.414054
2.611858	1.135316	0.508589	0.98126	1.476542	0.472672	-0.63461
3.109554	2.228999	0.514379	1.252314	0.880554	0.737935	-0.712395
2.45684	0.777748	-0.356507	2.248973	1.679093	2.60548	-1.02594
2.358315	1.712279	1.182316	1.541499	0.646036	0.359183	0.020547
1.449446	1.622246	1.028204	1.712279	-0.172801	0.684074	-0.247509
3.769018	2.123173	2.228999	3.002172	1.645845	0.773173	-0.420818
3.115466	1.733697	2.782638	2.782638	1.381769	0	-0.581368
1.541499	1.147326	0.506694	1.278666	0.394173	0.771971	-0.661582
1.220769	0.514379	0.626727	1.541499	0.70639	0.914772	0.248924
1.584738	-0.539274	0	0.626727	2.124012	0.626727	0.177705
1.66898	1.590347	1.398149	1.934843	0.078633	0.536694	-0.348173
2.561828	0.771422	0.865095	0.955128	1.790406	0.090032	-0.38249
1.952393	1.350839	1.366022	1.952393	0.601554	0.586371	-0.396589
1.695335	1.252314	1.110772	1.635172	0.443021	0.524401	-0.586501

2.124012	0.955128	1.366022	1.952393	1.168884	0.586371	-0.954027
1.683242	1.622246	0.183706	0.799528	0.060996	0.615822	0.066687
1.534899	0	-0.17738	0.692295	1.534899	0.869675	-0.001093
2.087262	1.278666	1.158641	1.308116	0.808596	0.149476	-0.051586
-0.093673	0.771422	0.085453	0.087348	-0.865095	0.001895	-0.442166
1.365203	1.569535	0.369579	1.110772	-0.204331	0.741193	-0.536947
2.123173	1.451466	1.635172	2.342707	0.671706	0.707535	-0.172661
2.248973	1.590347	1.451466	2.078193	0.658626	0.626727	-0.678417
1.158641	0.98126	0.347076	0.963621	0.17738	0.616545	0.316965
1.622246	1.147326	2.009465	1.252314	0.47492	-0.757151	0.093562
1.838685	1.147326	0.98126	1.695335	0.691359	0.714074	0.079572
2.611858	2.944686	1.635172	2.563103	-0.332829	0.927931	-0.360843
2.124012	2.009465	1.833915	2.944686	0.114547	1.110772	-0.820842
1.809043	1.220769	1.182316	2.221543	0.588274	1.039227	-0.900147
1.695335	0.297186	0.084242	1.569535	1.398149	1.485292	-0.14207
1.695335	1.364119	0.169695	1.590347	0.331216	1.420652	-1.322563
1.350839	0.963621	0.167894	1.220769	0.387218	1.052875	-0.676221
1.094968	1.220769	1.135316	0.98126	-0.1258	-0.154055	-1.256917
1.398149	1.904477	1.66898	1.683242	-0.506328	0.014263	-0.158079
0.347076	-0.192198	0.387218	0.430727	0.539274	0.043509	-0.867757
1.398149	1.398149	1.398149	1.182316	0	-0.215833	-1.071503
1.838685	1.350839	1.182316	1.590347	0.487846	0.408031	-0.273648
1.272349	1.464547	0.430727	1.712279	-0.192198	1.281552	-0.78355
-0.183706	0	0.192198	-0.508589	-0.183706	-0.700787	0.94285
1.068608	1.590347	0.811565	1.635172	-0.521739	0.823607	0.413188
0.424347	0.684074	0.770077	-0.084242	-0.259728	-0.854319	-0.258154
0.455032	0.257043	0.336999	0.500926	0.197989	0.163928	-0.854413
0.622926	0.684074	0.335788	0.865095	-0.061149	0.529307	-0.138863
1.635172	2.123173	0.644262	2.025486	-0.488001	1.381225	-0.148801
1.158641	0.865095	0.524401	1.333192	0.293545	0.808791	0.185643
0.514379	1.398149	1.068608	1.333192	-0.88377	0.264584	-0.177641
0.508589	1.491822	0.598621	-0.297186	-0.983233	-0.895807	-0.790535
0.440749	0.340695	0.356507	0	0.100054	-0.356507	0.734551
0.586371	0.356507	-0.440749	0.673727	0.229865	1.114476	-1.106122
2.124012	1.147326	0.514379	2.009465	0.976685	1.495086	-0.783752
0.79082	0.799528	0.083652	1.683242	-0.008708	1.599591	-0.42081
0.434018	0.271053	0.093673	0.443021	0.162964	0.349348	0.706645
-0.332829	0	-0.986381	0.552363	-0.332829	1.538744	-1.47891
0	0	0	-0.332829	0	-0.332829	#NULL!
#NULL!	#NULL!	#NULL!	#NULL!	#NULL!	#NULL!	-0.228843
