# Supplementary material for: Ownership Effect Can Be a Result of Other-Derogation: Evidence from Behavioral and Electrophysiological Studies
Source: PLoS One. 2016 Nov 4;11(11):e0166054. doi: 10.1371/journal.pone.0166054 (PMC5096666; doi:10.1371/journal.pone.0166054)
Supplement: S2 File — (PDF) [file pone.0166054.s002.pdf]

File	Fp1-self_p	Fp1-self_n	Fp1-other_	Fp1-other_	AF3-self_p	AF3-self_n
sub03	0.385	2.3766	1.9838	7.0171	-2.5457	-0.1857
sub04	-5.0741	-1.4528	-6.0854	2.7319	-3.3083	-3.351
sub05	-2.274	-0.0395	-3.7672	-1.2123	-3.9385	-2.3523
sub06	3.6672	-1.3359	0.3888	-1.8436	1.0476	-3.1749
sub07	-2.9647	-3.5847	-3.3668	-2.3181	-0.2269	-0.9755
sub08	0.117	-3.6395	1.1019	5.8371	-1.3325	-2.8881
sub09	0.284	-1.7143	-0.4008	-0.5609	-3.5441	-4.7679
sub10	-6.3124	-12.9964	-13.221	-3.065	1.5434	0.6829
sub11	-8.7262	-3.8867	-2.6142	4.1319	-3.2824	-5.6149
sub12	6.238	3.7979	-4.1162	0.8654	2.7106	-1.4943
sub13	0.0574	-2.3624	-2.3812	-0.4108	-1.7885	-3.1844
sub14	0.1313	-1.7023	3.7246	-0.8036	1.7411	-1.9312
sub15	-2.3561	0.8489	-2.0463	-0.3846	-2.1466	2.4108
sub16	-0.7097	-3.606	0.1002	1.7033	0.968	-2.8583
sub18	-2.4068	2.8844	-0.4398	-6.7534	-3.7907	-6.7461
sub19	8.6273	0.7131	6.2165	17.6394	-4.7211	0.4658
sub20	-4.2011	-1.4592	-5.0803	-3.441	-5.678	-4.4509
sub21	-1.6163	-0.8197	-1.6174	-1.116	-1.7286	-0.6465
sub22	1.457	-1.0624	-2.8349	-0.8526	-2.1904	0.9663
sub24	-2.2564	7.2919	1.0627	1.5107	-3.0389	0.0739
sub26	-1.0333	-2.0299	-0.1299	-1.0475	-3.726	-2.8312
sub28	-4.1051	1.404	-1.6366	-1.8797	-1.586	-0.8645
sub29	0.3552	3.0975	-4.3945	4.8785	-1.9784	-1.6558
sub31	-4.9371	-3.3228	-1.9569	-1.4725	-5.9597	-2.0041
sub32	-5.4338	-4.3252	-1.4251	-3.4772	-6.1076	-5.2292
sub33	-2.4283	-8.4057	0.7571	1.4238	-0.8159	-3.7018
sub34	1.1231	0.6372	1.8428	1.1444	0.8078	1.7007

AF3-other_	AF3-other_	AF7-self_p	AF7-self_n	AF7-other_	AF7-other_	Fz-self_po
0.1703	2.49	-0.7304	2.9614	3.7076	4.6022	-5.2151
-3.7843	0.6421	-4.2143	-3.1819	-4.1688	0.9161	3.2191
-4.0226	-2.7652	-1.9556	0.2794	-2.3216	-0.9356	-5.1308
0.0815	-0.6577	2.1501	-3.4779	-1.6162	-1.6884	-1.5891
1.5202	-4.8186	-0.534	-2.4583	1.0442	-2.6596	-1.5515
-1.5745	-1.6245	0.2074	-0.5372	2.7851	0.7337	-5.6361
-2.8711	-3.3454	-0.2357	-1.5895	-1.5001	-1.7301	-6.039
1.9701	-0.5016	-1.7756	0.3913	-1.9412	-4.495	0.8252
-3.2578	-1.5262	-10.0761	-2.2934	-3.9351	1.0342	-9.5383
3.1478	0.7162	8.2875	6.5437	-3.3707	0.1237	5.7811
-3.3826	-1.5908	0.9813	-2.066	-0.9809	0.3861	-4.7187
2.4333	3.6539	-3.3018	-2.1804	3.3896	-1.0829	1.1592
2.2838	1.9819	0.3983	1.7951	0.3537	0.7734	1.6649
0.0218	0.1836	1.3698	-0.7826	1.4459	0.9702	-1.6866
-2.9027	-0.0811	-0.0587	-1.1581	1.0616	1.3118	-7.8968
-4.01	-3.7177	6.2636	-5.5049	-2.3456	-1.847	-2.5846
-8.3811	-7.105	-2.023	-1.1638	-3.503	-3.1589	-3.4791
-2.9086	-2.4816	-2.0296	-1.8539	-1.443	-0.2332	-5.7504
-4.2967	5.6591	2.2038	0.5916	0.1612	3.4466	-6.4369
-2.4521	-0.2609	0.1885	8.2426	2.9816	0.4514	-3.3306
-0.195	-1.8726	-1.8904	-2.6731	0.0781	-4.608	-2.9205
-0.914	-2.9075	-3.1723	-0.1566	-4.2027	0.6514	-7.3837
-1.6429	-4.6354	-4.2655	-2.3084	-12.8822	-12.4949	1.9101
-2.2122	-2.469	-3.5094	-1.2733	0.3603	-0.3938	-2.5176
-1.5356	-4.0605	-2.6172	-3.0988	-1.0894	-3.0521	-7.3781
-2.9911	-4.7077	0.0975	3.2186	-1.004	1.7551	-3.4153
-2.5907	0.8813	1.7866	0.83	1.1127	1.7419	1.5941

Fz-self_ne	Fz-other_p	Fz-other_n	F1-self_po	F1-self_ne	F1-other_p	F1-other_n
-2.507	-0.7796	-0.3255	-4.4158	1.2798	-1.8442	0.5072
0.6366	-0.1807	-0.7007	-0.7724	-2.7087	-1.7728	0.4867
-1.746	-5.5616	-2.9961	-5.989	-1.0643	-4.0644	-1.9624
-2.0416	-1.3279	-0.1856	-0.6307	-3.172	-0.6002	-0.2562
0.9992	2.7753	-1.7019	-0.5337	-1.0306	1.9969	-3.4082
-8.2372	-6.2523	-7.5585	-4.1824	-6.1001	-4.0065	-4.3118
-4.3913	-5.6595	-3.3085	-6.1704	-5.2893	-5.9057	-3.1838
-0.4209	0.269	-1.9052	-0.1909	-1.5959	-3.3331	-1.9202
-2.0399	0.4766	-11.669	-2.0033	-3.095	0.3084	-6.1161
4.7053	1.7711	1.1892	5.3761	3.3895	1.0356	0.9344
-5.0314	-3.9999	-3.1922	-5.2913	-3.5806	-5.2165	-3.7014
2.2715	4.1323	2.8169	-0.1869	-0.7112	2.7352	4.1879
2.2985	0.0531	-0.0829	2.7659	0.343	1.639	-0.7611
-5.0982	-4.0212	-3.1062	-1.0971	-3.5353	-2.8159	-2.8359
-10.6284	-7.5009	-8.4162	-6.4867	-8.8365	-4.197	-6.4723
-2.0394	-1.9945	1.8451	-2.9861	-0.8673	-2.0116	0.0306
-2.2907	-6.9663	-5.5784	-3.3825	-2.6776	-6.9949	-5.9458
-5.047	-4.98	-4.961	-5.3941	-4.0385	-4.1738	-5.2024
-1.3444	-5.5139	-0.4929	-1.3517	-0.7427	-0.2293	0.0019
-2.0321	-1.5155	-0.2444	-2.9403	-2.1345	-1.1116	-0.7564
-3.3252	-0.8214	-1.6292	-3.28	-2.8888	-1.8342	-1.6941
-4.1704	-5.3342	-4.4216	-6.4913	-3.4685	-3.7292	-2.8627
1.7515	-1.3973	-0.5772	1.84	2.4628	-0.4282	-1.0618
-2.0657	-0.3704	-0.8087	-4.1227	-1.4412	-1.41	0.2594
-6.2856	-1.3239	-4.7866	-6.9394	-5.6863	-1.5205	-4.851
-7.3381	-3.5467	-2.3897	-5.7145	-5.5175	-6.6678	-5.3126
3.0165	3.6783	1.641	1.5881	3.0734	3.5257	1.728

F3-self_pos	F3-self_neg	F3-other_po	F3-other_ne	F5-self_pos	F5-self_neg	F5-other_po
-3.7354	-1.4293	-0.4597	1.643	-0.1502	0.6084	0.7737
-1.7941	-2.5814	-2.9462	1.2706	-0.1808	-0.9601	-2.6488
-3.5709	-0.333	-2.8449	-1.2964	-0.6281	1.209	-2.4216
0.0478	-2.232	-0.212	-0.1642	0.9591	-1.5736	0.1275
1.9865	-1.6068	-0.2003	-1.8226	1.4131	-0.1695	3.0809
-1.5165	-3.3676	-1.9389	-2.4502	-0.0575	-1.9527	-0.5488
-6.3423	-3.0361	-4.0937	-5.5575	-3.8734	-3.4624	-3.6465
1.9106	1.8364	1.392	-1.1665	2.072	1.732	1.6551
0.0438	-0.9777	1.2988	-2.8592	2.4428	-1.8056	-5.1024
7.0033	0.4783	-2.1429	0.2594	0.847	4.0968	2.46
-4.083	-3.4334	-5.0819	-2.5781	-2.9735	-2.3729	-4.0601
1.4536	-3.2805	-0.865	2.2923	-3.2324	-0.2984	0.1197
-2.4403	4.0064	4.0089	2.0285	1.3616	3.9838	0.4641
1.0379	-3.101	-0.9202	-0.2272	2.3772	-0.7487	-0.6888
-6.1655	-7.6582	-3.8909	-6.321	-3.1563	-1.8621	0.0188
-1.5365	-1.2432	-1.77	1.087	-5.5687	4.589	-7.0737
-2.1155	-2.3579	-6.9672	-5.747	-0.3513	-1.0114	-3.8632
-3.0308	-2.6245	-2.9917	-2.6517	-1.5102	-1.1741	-1.948
-3.6706	0.1606	-3.8679	0.6618	-1.5632	3.3877	-0.4674
-2.1807	-3.4208	-3.4307	-2.7951	-3.4244	-0.1978	-2.2402
-4.1379	-3.1719	-1.7195	-0.7904	-2.2338	-2.3388	-0.5652
-4.9224	-2.4001	-2.005	-3.073	-2.327	1.0836	0.0575
1.9597	3.0371	1.013	1.0569	-0.1456	1.5329	-1.9529
-3.111	-1.7179	-2.1704	1.4691	-3.1689	-0.4021	-0.8498
-6.4367	-5.2878	-2.0245	-6.6743	-2.0262	-4.3182	-0.9351
-4.0606	-5.1053	-4.4338	-6.1992	-4.7224	-3.0942	-4.6625
2.2208	1.8807	3.2608	2.1228	4.4603	1.0706	2.1252

F5-other_n€	F7-self_pos	F7-self_neg	F7-other_p€	F7-other_n€	FC1-self_p€	FC1-self_n€
3.8255	-0.5202	1.7203	-0.5506	1.4771	-4.3993	-2.6873
-0.0434	-2.1769	-2.5353	-2.5059	-0.1299	0.2079	-0.8302
-0.5028	-2.043	0.4369	-2.2418	-2.0996	-2.7975	0.3882
-0.0045	0.3305	-3.354	1.1251	0.591	-2.0023	-1.8427
-3.5602	-0.0206	-2.0308	1.4904	-2.893	0.4957	2.6103
-1.1046	-1.1575	-2.0362	0.4511	1.1252	-5.0617	-8.0814
-2.5973	-1.3444	0.1933	-0.3699	-2.5242	-6.5626	-4.561
0.37	0.5633	1.218	0.7817	-1.1293	1.5964	1.2037
6.3115	-0.9209	-1.1666	-1.7793	6.4641	-0.3379	-9.845
4.558	4.6243	8.2597	-6.5678	5.1905	5.7799	4.269
-2.6963	-1.5777	-2.1791	-1.7141	-1.0709	-5.0019	-4.6832
1.9878	-3.1078	-0.4263	0.1075	1.9695	0.7082	0.4468
4.0126	2.2656	2.1906	-1.6882	1.9427	5.216	5.1332
-0.4576	1.0033	-1.7967	0.5231	0.9245	-0.5151	-2.5612
-0.7269	-2.0165	0.266	0.0332	-0.0719	-6.7491	-8.4173
-3.9928	-5.7037	4.641	-7.3223	-4.2184	-1.9177	-0.9717
-2.8632	0.8651	-0.1717	-2.2555	-1.4479	0.0184	-1.3943
-1.231	-1.5219	-1.3146	-1.4344	-0.9607	-5.7511	-3.3569
3.1548	0.839	0.4754	-1.4025	0.1834	-2.0077	-0.719
0.1818	-2.8224	-3.4546	-4.4488	-1.0857	-2.1279	-1.0751
-0.9953	-1.4208	0.8467	1.5701	1.003	-2.1869	-1.8639
-2.5427	-0.8748	1.659	-1.4064	-1.4594	-4.49	-1.0407
-0.855	2.0287	4.5225	0.9379	0.3428	1.079	1.4869
-0.6589	-3.1311	-1.1257	-0.0709	0.672	-2.2458	0.3054
-3.91	-2.2279	0.2706	-1.6163	-0.4418	-5.9531	-5.1519
-5.8368	0.4093	-4.9938	0.4276	0.573	-2.7678	-2.8654
0.3587	2.3698	2.2857	1.3381	-1.1895	0.8541	2.8093

FC1-other_p	FC1-other_n	FC3-self_po	FC3-self_ne	FC3-other_p	FC3-other_n	FC5-self_po
-1.4645	-1.0895	-2.4043	-0.9269	-1.5568	1.1001	-1.6986
-0.997	0.9266	-1.0216	-1.6394	-1.4573	-0.0574	-1.6106
-3.3818	-0.9095	-2.2764	2.356	-1.9637	-1.4162	-1.0262
0.4073	-1.2873	-0.1984	-0.9091	0.6749	-0.1525	-0.245
3.2231	-3.4132	1.6322	-0.5871	2.8057	-0.4684	2.1343
-6.5838	-6.9369	-1.6289	-3.6657	-2.6677	-3.5658	0.5479
-4.3814	-3.3274	-6.1401	-4.4438	-4.8128	-4.6397	-2.0484
1.738	-0.5987	2.1271	1.8769	2.4355	-0.5069	1.8059
-1.6271	-6.6504	1.3752	-0.4746	1.4877	-0.1021	0.3483
1.4138	2.9171	6.2361	1.7892	-0.3275	5.5859	4.4738
-5.0081	-3.2213	-5.118	-4.7759	-5.5442	-5.1229	-4.076
2.0702	3.3548	-0.2874	-2.4033	1.8651	2.2555	-3.0157
2.7234	1.7449	3.4248	4.301	3.1417	2.4971	-0.9026
-2.8739	-2.5099	0.9545	-1.0372	-0.2573	-0.1473	1.7646
-3.2043	-6.9854	-5.5456	-6.7384	-3.7441	-5.93	-2.7178
-1.8077	2.2913	-2.0793	-0.3651	-0.4838	-1.0574	-0.6389
-3.8116	-3.8203	0.959	-0.6771	-3.8763	-4.0724	2.6608
-3.395	-4.3527	-3.3812	-2.1914	-2.2428	-3.0594	-1.1539
-2.6393	-0.6499	-4.2179	-0.8152	-2.4225	-0.3129	0.8889
-1.3881	-2.8554	-1.2106	-1.4288	1.4048	-1.1997	-0.9277
-0.2794	0.2082	-1.985	-2.085	-0.6767	-0.2288	-0.7519
-1.4605	-1.9115	-2.9319	-0.5734	-1.0937	-1.0016	-1.827
-1.4274	-0.7439	2.975	2.4042	-2.3731	0.5395	2.4901
-0.1363	0.2998	-1.205	1.1173	-0.3777	0.552	-1.0674
-0.6239	-4.6339	-5.7738	-4.6663	-0.6643	-4.322	-4.236
-3.1372	-2.6806	-3.8915	-6.8545	-10.3629	-5.7293	-0.9386
3.6056	0.3085	1.5962	1.8826	2.5931	-0.6441	1.9613

FC5-self_ne	FC5-other_p	FC5-other_n	FT7-self_po	FT7-self_ne	FT7-other_p	FT7-other_n
2.2007	-0.2396	2.6102	-1.6298	0.477	0.1442	0.9113
-2.1412	-2.1794	-0.4251	-2.3906	-2.48	-2.6469	-0.2471
0.8268	-1.9534	-0.9532	-0.9967	-0.0676	-1.8589	-1.5611
-1.2673	-1.3265	-0.0271	-2.2276	0.1387	-0.1826	0.6592
0.6078	4.0051	-1.1772	2.0919	-2.476	2.4182	-1.4504
-1.79	0.3264	-0.1617	-1.216	-1.8364	-0.7171	1.2419
-3.4134	-2.6471	-2.2111	-1.1458	0.056	-1.7165	-4.4607
2.0029	2.0417	-0.4125	1.1242	1.2855	1.2931	-0.549
-2.4945	1.2277	0.358	-0.4407	-1.0021	1.7274	0.0242
3.7638	1.7468	2.7237	4.3316	5.0349	0.5634	3.1167
-5.0071	-3.6194	-2.504	-1.9102	-1.5885	-1.6279	-0.5944
-1.2293	4.7157	2.6375	-4.2448	-2.8032	1.6337	3.794
4.4582	0.9051	4.5622	1.3128	3.5027	2.6463	4.3353
-0.5062	0.8336	1.0732	1.3441	0.1246	1.1393	1.0665
-1.9721	-0.313	-2.5096	-1.7527	0.0542	-3.6977	-2.8012
0.997	-0.1185	0.4244	-2.3021	-0.1026	-1.5886	-1.3727
0.711	-1.4575	-0.4353	3.386	0.9523	-0.2241	0.6449
-0.7561	-0.822	-0.7165	-1.0803	-0.8736	-1.0311	-0.8149
-1.9081	1.485	1.431	-0.1698	1.3905	1.5211	-0.3709
-0.7465	0.1835	-0.0187	-0.9451	-0.6049	-0.3817	-0.0337
-1.0268	0.0496	0.0322	-0.6854	-0.6209	1.0746	1.3538
0.7962	-0.4889	-0.0916	-1.805	0.9447	-0.4909	0.0978
4.4904	1.631	1.342	3.5293	1.6444	-7.1669	-0.1016
4.0282	2.079	4.5707	-1.2589	2.7248	2.6486	1.187
-2.8119	-0.5738	-3.389	-1.5817	-1.9657	-0.1602	-1.3531
-4.1675	-3.615	-2.2851	-1.9216	-2.214	-3.4364	-1.9617
2.2979	2.4242	1.0437	2.1073	2.4839	1.7024	0.8916

FCZ-self_p	FCZ-self_n	FCZ-other_	FCZ-other_	C1-self_po	C1-self_ne	C1-other_p
-6.0372	-3.1887	-1.7324	-0.8486	-4.0301	-1.0741	-0.5068
3.9126	0.5437	0.4023	1.4174	4.077	2.1092	2.8078
-4.1498	0.7763	-4.9393	-1.6382	-1.5672	3.0036	-1.3953
0.0197	-0.6459	-1.1585	0.3974	-0.4721	-0.1514	0.411
0.0687	3.4272	3.8503	-1.5805	3.6293	4.8351	5.9766
-6.929	-10.1466	-8.3116	-9.3058	-2.8757	-5.382	-4.3534
-6.5527	-4.1706	-5.8279	-4.4556	-4.2973	-2.2906	-1.9332
1.4114	0.6438	2.2161	-0.6578	-1.5874	-2.0631	-3.6487
-2.9035	-4.12	-1.1103	-5.102	0.7466	1.3128	3.0714
4.2403	3.0208	3.8483	3.4054	8.7965	7.253	5.6573
-3.2739	-5.4295	-5.281	-3.9059	-2.8414	-3.7182	-2.5556
3.5061	-1.0471	5.4464	-0.146	2.2137	0.0156	2.888
3.4043	3.9514	1.234	1.6141	7.2375	4.8433	4.1621
-2.6124	-4.2981	-5.2451	-2.8522	0.3511	-1.6628	-2.2212
-5.37	-10.2719	-5.0489	-10.3052	-3.398	-4.8765	-1.5307
-1.9781	-1.3466	-0.64	3.1751	-0.0821	0.955	3.2503
-0.5107	0.0846	-2.5984	-5.1398	3.9178	1.3311	0.5423
-5.9266	-4.3317	-4.4169	-5.9136	-2.7415	-1.974	-2.5685
-5.02	-1.1931	-4.0771	0.0489	-1.5292	-0.6918	-0.1023
-3.5897	-2.2316	-0.7746	-0.7086	0.0919	-0.187	-1.1552
-2.439	-1.4381	-0.0804	0.8503	-0.2823	0.2118	1.6856
-4.2817	-1.7124	0.0658	-0.7715	-1.5837	2.5136	3.8977
1.9663	0.524	-1.1593	-0.677	1.7668	2.1443	-0.0241
-1.861	1.3187	0.2171	0.6866	-0.4186	2.5177	1.5974
-5.3858	-3.7881	0.2415	-3.1086	-1.7478	-1.931	2.1387
-2.3767	-2.8744	-1.7447	-2.4792	-0.4277	-1.3303	-0.4552
0.563	2.4168	3.4243	-0.0894	-0.2428	0.6581	2.5464

C1-other_n	C3-self_po	C3-self_ne	C3-other_p	C3-other_n	C5-self_po	C5-self_ne
-0.5657	-2.4255	-0.0008	0.3634	1.2128	-1.2517	1.4493
0.2617	-0.7012	-1.006	-0.6103	0.2836	-1.5606	-1.6616
0.5781	-0.7679	3.3654	-0.7708	1.1918	-0.464	2.2271
1.1185	0.1149	-0.4512	-0.018	0.5731	2.148	-1.655
0.0611	4.3203	2.9381	5.6662	-0.3112	1.3265	0.9755
-4.6538	0.3599	-0.907	-0.5266	-1.1457	1.4869	-0.2506
-0.4619	-4.3862	-2.6161	-2.2906	-2.8669	-2.845	-0.9166
-3.4639	2.7348	2.8791	3.4381	0.5361	1.867	2.7566
-1.9164	3.0862	2.2985	3.7413	0.2806	1.7499	0.5731
0.8669	5.6043	4.0829	2.836	2.3718	2.1675	2.5809
-1.3154	-3.4127	-2.8903	-4.7243	-2.7927	-3.2909	-3.1017
2.9912	0.8848	-0.0514	3.5239	2.1912	0.2728	2.3054
3.0658	5.845	4.6265	4.8104	1.8768	4.5038	3.3172
-0.5195	2.0198	0.1562	0.1283	1.3472	3.1226	0.5994
-5.6722	-1.7558	-2.5869	-0.078	-3.2385	0.0688	-1.2111
3.5214	3.8067	1.9345	3.0897	1.3316	1.1552	2.2501
-0.1267	4.0395	2.6108	0.2687	-1.1504	4.7019	2.8728
-2.2897	-2.3055	0.7429	-2.1631	-0.7761	-0.4041	0.7528
0.6264	-2.3026	-3.1201	-2.1395	3.1091	0.8852	3.2726
0.1625	0.0172	-2.1985	2.2413	0.1214	-1.3462	-0.9812
2.229	-1.0392	-0.0759	2.5324	0.0638	1.2633	0.5418
0.3122	-1.9712	4.3204	2.1863	-0.0286	0.2483	1.6675
-0.159	3.0232	4.2763	1.2048	1.6614	3.4908	7.897
2.6304	1.8144	1.055	-0.5146	0.6271	-0.0543	2.6446
-1.1525	-3.5322	-1.9934	1.7434	-2.4705	-2.6501	-0.7902
-1.9732	-0.3224	-0.0387	-0.1145	2.9484	0.7233	-0.2309
-1.9896	-0.9685	0.3186	2.0434	-2.3509	0.3189	1.4277

C5-other_p	C5-other_n	T7-self_po	T7-self_ne	T7-other_p	T7-other_n	CP1-self_p
1.8718	1.975	-0.1037	0.2435	0.1623	1.6181	-2.4184
-1.8863	0.1887	-0.6097	-1.4105	-1.3826	-0.4734	1.1385
-1.3629	-0.6027	-1.0864	-0.1512	-1.1629	-1.8395	0.6502
-0.9815	0.0273	-3.6795	-0.9541	-1.0807	1.7906	1.0995
1.9283	-0.4672	2.8344	-0.802	2.5116	-0.8599	5.3636
1.3991	1.258	1.4324	2.1329	1.4074	0.727	0.5767
-0.655	-2.9329	-1.9452	0.1333	0.9621	-3.9956	-2.3276
2.6869	0.288	1.3181	1.7866	1.7303	-0.1296	4.8412
2.5941	2.4837	4.6184	-1.3529	-3.2054	5.5662	3.2397
1.5659	3.4851	2.483	3.3059	2.2037	8.8128	9.2472
-3.301	-3.0806	-1.5632	-2.819	-1.4473	-1.2808	-0.956
0.9954	1.5708	-5.0128	-0.0475	1.3343	4.7515	1.7745
0.3409	4.1095	1.5358	3.7899	1.2303	2.9377	8.3656
2.9989	1.0829	2.2903	-0.7482	2.2647	2.3646	1.2933
0.3302	-3.7506	1.0821	2.1235	0.5487	3.4324	0.5055
1.4966	1.0632	-2.9182	5.2947	-3.9236	-5.095	4.773
2.3731	2.3381	5.0372	1.7383	2.1444	2.2945	5.1576
0.3216	0.0921	-0.3923	-0.2814	-0.5203	-0.4152	-0.7246
1.4037	3.9298	-4.867	3.6096	2.3162	3.3333	-2.1767
-3.1322	-0.6302	0.8668	1.2347	0.8874	0.0384	-0.201
1.2358	-0.2873	1.3579	0.5774	1.1446	-0.2794	1.4169
0.0361	1.1835	-0.0097	1.6264	0.1439	1.1227	3.2896
-1.233	0.74	4.1675	2.0207	-2.4417	-2.1098	6.2833
2.2578	2.0959	2.8729	2.9021	3.5781	2.2957	1.0521
1.9526	-2.3705	0.4591	-0.7197	2.9818	-0.6731	-4.6314
-0.8822	0.1236	-0.0167	-1.2304	-2.2447	0.2862	-0.4333
1.8874	-0.429	3.5049	2.1095	1.7076	0.4019	-1.0398

CP1-self_n	CP1-other_	CP1-other_	CP3-self_p	CP3-self_n	CP3-other_	CP3-other_
1.2076	0.6311	0.5345	-1.0861	1.2245	1.4249	1.4334
1.0345	2.3719	1.7707	-1.4011	0.3656	0.6831	3.8665
6.9816	-0.5413	3.2442	0.4666	5.5972	0.7033	1.8858
1.5667	0.9401	1.7725	0.4737	0.8806	0.8963	1.5673
9.0054	7.881	2.7587	3.2824	7.8147	6.9416	0.6432
-1.1317	-0.8594	-1.636	2.6721	2.6372	2.813	1.4344
0.2721	-0.5333	0.7596	-1.855	0.1099	-0.4623	0.3971
5.0318	5.3564	2.9976	4.1339	4.9097	4.1661	1.8696
2.7408	4.7587	2.6661	4.0107	4.1106	5.0745	1.632
9.6538	8.0432	3.7604	5.5509	4.0143	3.1187	4.5528
-2.0838	-1.1416	0.3282	-0.9894	-0.6656	-1.7056	-0.5316
1.6782	2.853	3.7339	-0.2091	1.6923	5.702	4.8456
6.1861	6.4586	4.4238	7.1039	5.981	6.4936	4.3821
-0.4002	-0.1747	0.806	2.6696	1.8071	1.6531	2.7956
-2.5965	-1.6722	-5.2796	0.4686	0.7602	2.4883	-1.2188
5.0117	7.8949	7.0557	5.3664	4.6175	6.7554	4.7287
3.0543	4.376	0.2643	5.8846	4.3915	4.5815	1.2586
0.8268	-0.1443	-0.5397	-1.2973	1.2485	-1.4702	2.1393
-0.4795	-0.2819	0.7429	-1.7202	1.7933	3.007	0.8414
-0.9001	0.1771	2.0789	1.2715	0.5868	1.2027	2.6131
1.4998	3.4111	3.52	0.4877	2.2392	3.399	3.1292
5.9234	6.6975	4.1619	4.0983	5.3666	3.1464	4.4928
4.5058	0.0437	-0.6006	4.1203	6.2299	2.9296	2.7488
4.4323	2.5948	4.2184	5.9576	5.2984	2.7975	2.6977
-2.9169	-0.9982	-4.4767	2.3257	1.3463	4.0294	0.1897
3.1115	6.262	6.4073	7.2153	0.2039	2.9582	6.0169
0.0741	1.6173	-3.3205	-2.0389	-0.6051	0.3598	-2.9711

CP5-self_p	CP5-self_n	CP5-other_	CP5-other_	TP7-self_p	TP7-self_n	TP7-other_
1.3439	0.4183	-3.146	3.0304	0.2761	2.1833	-0.1239
0.9072	-0.9556	1.0957	-1.9258	-1.7441	-0.7532	-1.3008
-2.2208	4.1458	-0.8065	-0.5645	-0.643	1.7603	0.6333
-0.4794	3.2653	-0.9398	-0.7323	1.456	-0.6156	0.6526
4.3984	6.8262	7.3412	1.6749	4.6251	2.8558	6.5012
3.2882	3.0218	3.5852	3.6795	2.0948	1.5643	2.4687
-1.3167	0.3711	0.5838	-0.5364	1.5065	0.7601	0.6332
3.0504	3.2513	2.8365	1.0758	1.7019	2.0286	2.0034
0.7397	2.6765	5.0101	3.3739	3.914	1.838	0.8319
2.9078	3.7882	4.9242	1.1113	1.4773	-2.2306	0.3136
-2.8624	-0.2541	-0.4435	-0.5326	-0.9401	0.0991	-1.6536
-3.3936	0.509	2.6875	3.1707	-5.1555	3.6544	-0.147
1.0293	5.8203	7.0657	2.8716	2.9615	3.9327	0.6168
2.693	0.279	2.1117	4.1275	0.7839	0.4372	0.3105
1.7473	3.6185	1.551	-2.6831	1.4381	2.2038	-0.1462
3.2157	4.657	4.0443	4.5044	-1.6396	5.3635	-0.3658
6.4959	4.4387	2.8226	2.7794	6.5492	3.0687	3.7824
0.3713	0.6236	0.9587	-0.3123	0.1111	0.1008	0.0616
1.2165	0.9718	-0.4122	3.4733	0.2057	-0.3048	-1.6303
1.1295	1.3493	1.5321	2.1223	1.2865	2.2958	1.6446
1.2046	1.4474	2.7948	2.4411	-0.7314	-0.7395	-0.3907
1.7711	3.8421	2.7653	2.6589	3.159	4.4964	3.718
4.1009	7.2343	0.6113	3.7475	1.0627	2.1107	2.0172
2.9294	4.4357	3.1676	4.1396	2.9357	4.3888	3.3609
0.2483	-0.1896	3.7738	-1.4461	1.7573	2.3107	1.8469
1.9473	0.5836	0.8869	1.9517	1.6342	-1.6169	-1.3739
0.3782	0.9033	1.4132	-1.3534	0.4347	2.5987	2.7017

TP7-other_r	CPZ-self_po	CPZ-self_ne	CPZ-other_p	CPZ-other_r	P1-self_pos	P1-self_neg
1.7409	-1.9734	1.3124	1.0131	1.0493	1.4163	4.1538
-0.255	5.0473	5.7444	4.5637	-0.2148	5.0758	6.2191
-1.144	1.1415	6.7746	0.5308	3.3394	3.1624	9.5515
0.4044	1.508	2.8049	1.7027	2.6872	2.05	5.6777
0.5752	6.4171	7.6101	6.5403	1.2392	-2.9037	-1.851
2.8884	-1.6756	-2.0947	-3.1894	-1.2905	1.9348	1.1305
0.0871	-1.3003	1.2709	0.1988	1.1873	-0.1098	1.9812
0.4849	5.3973	5.8302	6.0531	3.9851	6.3372	6.962
6.3671	4.794	3.321	5.5364	2.0153	6.8253	6.6724
-1.4153	10.5344	10.0614	7.5315	6.8116	8.6554	9.6191
-2.0311	-0.7974	-1.6544	-0.4946	0.941	-0.146	0.9967
0.8205	0.2995	0.2495	3.0824	3.2443	-0.9879	3.9762
0.2221	8.3635	6.7945	6.598	4.6081	7.8229	9.3038
0.9829	0.0396	-1.23	-1.3275	0.3578	2.4217	-0.8533
-0.2566	0.1449	-1.85	-0.2202	-4.0403	2.4298	1.2759
-0.7574	5.4998	6.1182	7.7476	8.529	7.5475	8.4314
3.1921	5.393	3.2464	4.4861	-0.2761	7.9411	6.8462
-0.1751	-1.1563	0.85	-0.6588	-0.8302	5.8791	4.8631
2.6238	-1.9984	-0.7268	-0.7096	3.1915	0.1163	0.618
0.4801	-0.2619	0.3629	1.1603	3.066	0.7624	1.7771
-0.5499	1.5224	2.9393	3.8112	4.6883	4.7372	4.0391
-1.2694	4.6251	6.9853	8.7264	5.8657	7.0825	9.2227
0.7009	3.3281	4.8199	1.7478	1.5051	3.5207	6.0085
4.5657	1.1102	4.1258	4.0896	4.0292	2.9649	5.1651
-0.2949	4.8172	3.3983	5.8003	3.5459	6.652	3.961
2.495	-0.2786	1.4941	4.6311	3.4759	1.9678	1.9469
0.6615	0.6309	2.7602	3.0433	-0.1293	-1.1418	0.4139

P1-other_p(	P1-other_n(	P3-self_pos	P3-self_neg	P3-other_p(	P3-other_n(	P5-self_pos
2.0382	3.339	1.5743	6.4299	-3.4514	3.814	1.6604
2.8461	1.4155	5.438	3.2048	5.498	3.1433	1.3017
3.851	4.9024	3.2434	7.9102	1.7036	3.8587	0.3042
2.9821	2.1144	2.0073	7.6338	2.4125	-0.3941	2.3362
1.1985	-3.6673	8.2931	8.2025	10.3083	3.1792	6.1519
2.0622	2.5122	4.5998	3.9378	4.4546	4.1507	2.8305
2.8652	2.1953	-0.2977	2.7818	2.1209	1.7817	1.7088
6.8636	4.6692	6.0733	5.468	5.7701	3.8632	4.3219
8.7511	3.9272	7.1525	3.7774	6.2113	4.342	5.1769
8.3474	8.0166	5.6101	3.3068	5.9704	4.9238	3.3302
1.0698	1.3211	-0.1968	1.9691	1.3761	1.2202	0.5809
2.3785	5.9007	-0.4978	2.7719	1.7525	5.0838	-3.1494
4.4342	8.7197	7.9709	9.3101	4.3305	8.6893	11.0364
0.6938	3.0921	2.0968	0.0365	3.5065	3.3958	2.2759
1.395	-1.8035	2.4259	0.5566	-0.3615	-2.8671	0.895
10.4301	9.9318	7.8662	9.0159	9.2436	4.7343	5.7607
7.7683	5.2926	6.7246	5.8064	7.377	2.4175	8.2921
1.4966	2.1473	1.3845	2.4562	0.8549	0.5211	0.8579
0.0025	3.0309	0.3651	0.734	-0.645	2.7003	-1.622
1.8926	2.8839	2.5224	2.5773	1.865	3.9268	2.5975
5.4087	6.0243	3.8815	3.3407	3.8826	4.2345	3.7418
8.3186	7.1761	5.6753	7.8097	8.8333	5.6543	3.8666
5.0026	3.1402	1.7395	8.283	4.8659	2.9595	6.4079
4.4921	4.3831	2.8753	4.4801	4.3769	5.9968	3.8755
7.1561	3.6897	7.8074	5.0084	6.6167	4.6881	5.0302
3.6093	1.8238	3.2821	1.2912	-0.1082	2.9613	4.9023
1.9198	-1.6235	0.9706	0.5987	3.1047	-0.6116	0.8449

P5-self_neg	P5-other_po	P5-other_ne	P7-self_pos	P7-self_neg	P7-other_po	P7-other_ne
2.8438	2.0018	2.7569	1.7369	3.2565	1.6184	2.6455
-0.0342	0.4986	0.4637	-0.6434	1.2223	-0.0238	0.7356
5.8517	-0.1119	0.8779	0.6428	3.0442	0.0399	0.2876
7.2769	3.7993	-1.5334	2.097	2.5044	2.3267	1.5786
6.7214	10.7491	5.2839	5.8728	4.0825	8.2963	0.5642
4.1281	5.0341	6.5656	4.0078	2.5948	3.8502	4.1831
1.9599	1.6496	2.8201	3.4717	1.0613	0.9769	3.0753
4.7954	4.1697	2.6581	2.1166	2.8262	2.6636	0.6899
6.5605	6.0868	3.132	6.7173	2.2605	1.2572	4.9568
5.3146	6.0292	4.2693	1.8026	2.3683	3.3805	2.8178
1.5597	0.1125	1.4643	0.876	0.5561	2.0101	2.9595
3.0757	-0.8287	2.6182	-6.6843	2.6356	-1.5898	4.1987
2.531	0.8615	2.0794	6.3284	2.9458	-2.8885	0.5548
1.583	1.7035	2.4751	1.9235	1.1547	1.3511	2.2032
4.4143	3.7626	0.241	0.3132	2.0091	0.5635	0.4686
7.0715	7.8917	5.8953	3.5686	6.0831	2.5366	5.7289
7.086	7.8436	4.9264	7.7991	5.2414	5.471	4.7573
2.5173	0.7968	0.5728	0.0015	3.0015	2.1425	0.8264
2.6851	0.4501	0.2656	-2.1941	-0.1996	-1.0852	3.4405
2.9142	2.2136	3.2131	2.5467	2.7199	2.2153	1.8425
3.3439	3.7317	4.1147	3.7101	3.212	3.699	4.0589
5.6989	5.3223	4.8826	2.2836	3.4914	4.2535	0.9665
8.2535	4.6037	4.7926	5.4494	7.6691	3.1559	4.4067
3.2403	3.6094	5.9149	0.4525	5.218	4.8021	5.7215
4.2229	4.4894	1.3755	2.8136	0.761	0.6669	0.1728
1.728	-1.5451	1.9279	-0.0688	-0.4996	2.8437	4.858
2.0435	2.1622	0.0299	-0.1689	2.4163	2.3363	0.0133

P03-self_p(	P03-self_n(	P03-other_I	P03-other_I	P07-self_p(	P07-self_n(	P07-other_I
2.8461	4.8163	3.921	3.9487	3.2216	3.4578	2.4619
4.1591	2.3137	2.4829	4.0563	2.1061	0.1627	1.8824
5.3255	9.6783	5.7557	5.3626	1.8168	6.9755	2.7653
-0.0953	9.2161	3.4461	-1.1569	1.0714	3.2789	1.9863
1.6551	0.619	2.7406	1.2531	8.0424	3.239	4.7214
3.058	3.7786	5.9983	8.5861	1.9957	2.9609	5.2285
3.7098	2.4527	3.6563	4.2294	5.9992	-0.1721	1.5055
6.2564	7.0074	6.4411	4.9624	3.62	4.3922	4.0936
7.4287	5.468	8.6492	2.2386	6.3605	6.8793	4.5013
6.9132	7.2669	6.885	6.7817	1.068	0.4788	3.2072
1.0831	2.991	2.1554	2.5714	1.3811	2.3557	1.353
-0.3092	-0.2844	-7.7169	6.2047	-3.7686	-0.4649	-10.2383
10.14	5.392	2.4247	3.443	6.5053	5.4215	-1.0766
2.6358	0.924	2.1783	4.1912	1.1959	-0.1616	0.9958
1.5052	2.8819	0.9793	-0.7016	-0.1057	4.1915	1.5557
11.3511	12.1995	12.5753	8.6053	5.8938	8.9367	8.6369
9.2407	6.8716	7.2904	5.8334	8.4864	5.9413	6.922
3.1084	1.5554	2.7648	1.4408	2.7874	2.9291	3.3951
2.8876	1.6979	0.6323	6.1089	-1.3368	2.5152	0.4814
3.0299	3.9815	2.4558	3.2704	3.6959	4.3413	3.2336
4.6378	4.2604	4.4658	4.2699	3.8729	4.3975	3.9165
6.6834	9.8497	11.5653	5.9759	3.1994	4.9431	4.2069
2.5975	8.9197	2.8989	4.0056	3.0811	7.3488	0.3819
1.8367	3.1047	6.0412	4.3801	2.5576	4.2498	1.469
8.2522	4.9728	6.9593	3.4111	5.1511	3.4372	3.6568
0.7008	0.1261	0.3542	1.5187	4.8116	0.7122	3.6399
2.0731	1.1094	0.8694	-1.6238	1.0647	3.8628	2.9978

P07-other_r	0z-self_pos	0z-self_neg	0z-other_pc	0z-other_n	01-self_pos	01-self_neg
3.0085	3.43	5.706	2.4933	4.9402	4.4149	4.6177
0.0925	0.3458	1.4375	2.5613	1.7026	6.9126	3.2567
1.6653	4.1683	7.7331	3.9249	3.9866	5.4587	8.54
1.2426	0.701	2.2057	0.4313	0.1132	0.8758	2.4403
5.2718	4.1863	4.0112	4.7287	-0.8329	4.2943	7.6169
7.2914	-0.331	-3.4282	-1.9499	-1.2465	3.753	2.8138
4.9283	4.1217	3.7945	5.0708	2.0445	3.4194	2.7365
2.415	6.1188	6.6768	6.3347	4.0915	5.8335	5.5349
3.8958	8.6089	7.9872	4.5105	1.8097	5.7464	5.1179
1.4864	2.2407	2.2624	2.93	3.6639	3.095	2.4248
3.2767	-1.2815	0.9089	1.1876	1.023	0.0958	2.5817
-0.2267	-3.5213	0.4532	-7.9636	1.5636	-5.5268	-0.703
0.9247	2.7678	1.8688	0.9665	1.7703	-0.1856	2.7527
1.2243	0.0372	-0.8866	-0.8765	0.701	1.1968	-0.1137
-1.4014	2.9605	1.6559	-0.3962	-3.1392	1.7509	2.3541
5.1332	5.7651	5.8817	7.1275	4.1497	5.9698	9.4368
5.5963	5.6834	4.6094	5.2415	4.3564	7.4589	5.5749
-0.3182	-3.8306	2.0179	5.6956	-2.4677	-1.5473	1.4099
1.6711	-0.3746	-1.8983	0.4269	0.1774	-0.8862	2.3204
2.3865	3.0791	4.0868	2.6275	2.5517	3.7108	5.519
3.4599	5.9073	6.0877	4.7542	5.3192	3.9372	4.3806
3.9473	4.3245	4.9331	6.8712	1.9585	-0.4426	8.289
0.9714	3.6888	6.4399	2.6222	3.8828	2.1495	5.21
2.4288	-0.6328	0.8493	2.1075	0.5727	0.949	3.071
3.7853	3.9005	1.6011	1.7977	2.4726	4.7897	2.1483
6.1556	1.7874	4.9308	5.9831	4.3218	2.0718	3.0698
-1.7531	-0.4889	1.3227	-0.9441	-0.1477	0.3756	3.4181

O1-other_p(	O1-other_n(	HEOG-self_]	HEOG-self_1	HEOG-other_	HEOG-other_	Fpz-self_p(
5.3433	4.867	3.3195	1.4889	6.6116	8.081	-7.6952
5.5761	0.6246	-5.8653	-3.2519	-1.6016	-3.2521	-0.6221
5.4016	2.1543	-2.2742	-2.1812	-2.066	3.3208	-5.6915
0.6387	0.8101	2.6485	0.3943	-0.3001	-3.8567	2.1962
10.2563	3.4272	-1.4138	-1.404	-2.9381	-2.1941	-1.9976
3.6919	4.5814	0.7131	2.0647	1.8168	-0.4647	-4.0963
2.4885	5.6868	-4.9208	-2.5167	-0.5598	-3.9245	26.42
13.3117	4.3088	7.8298	6.1751	9.0449	9.6769	-0.3881
3.7469	4.5595	-2.2719	0.6377	-8.571	-0.6446	-6.9045
3.1843	3.7449	-0.2714	-4.3108	-4.8782	-4.3375	2.3977
-0.1087	1.6541	-1.7977	0.2985	-0.5451	-1.4581	-7.6704
-8.4307	0.7155	2.001	1.0234	-1.3821	2.2261	3.4257
1.6105	0.3433	-3.9609	-3.9519	-1.1191	-1.7696	-3.5672
1.0525	1.222	-1.1275	-3.7339	-2.2528	-3.4319	-3.5065
-0.5493	-3.2222	-1.2222	-6.4805	-2.1443	-6.1885	-7.8136
7.6125	2.5722	-1.6964	3.6676	1.2889	-5.2258	-4.1005
6.6845	5.3385	4.213	1.2907	1.6217	5.0841	-9.6718
0.0913	-4.1613	-2.3026	-5.4774	-1.9512	-3.4223	-0.7032
-0.5617	1.5557	-0.6345	-0.9051	-0.5064	2.6227	-2.8725
3.5914	3.5013	0.5714	6.7371	-1.105	1.7235	-4.1755
3.9328	3.4121	-4.9535	-2.1597	-3.803	-3.4908	-5.8552
5.6185	3.9576	-2.8071	1.9325	-1.9609	-0.2347	-3.6069
-0.4611	2.5374	-7.2016	-3.4052	0.3883	-0.0191	-1.4165
1.7027	2.796	-2.0172	-4.5302	-1.6678	-6.0744	-4.8157
4.1327	3.4103	-1.3601	-2.5101	-1.0841	-2.2326	-1.9406
4.1985	2.609	-0.0738	1.847	-0.2757	0.7785	-2.1065
1.8327	0.1925	2.4016	-1.4961	1.4334	-1.2672	-1.4831

Fpz-self_n	Fpz-other_	Fpz-other_	Fp2-self_p	Fp2-self_n	Fp2-other_	Fp2-other_
-1.1884	-4.9656	-4.513	-3.6748	0.5252	-0.6221	2.0282
-4.2027	-1.4025	-1.5351	-1.7347	-1.6342	-3.6106	1.2114
-5.4509	-6.9506	-3.6921	-4.6223	-3.4742	-6.4513	-3.0608
-1.4135	-3.5466	-2.4853	0.4073	-0.7978	0.771	-0.7311
2.4141	1.9402	-4.7977	-1.9724	2.4153	1.9447	-4.8071
-1	-0.21	9.6585	-4.1822	-3.4902	-1.9026	2.1193
5.5239	-0.1487	4.8759	-0.1345	-0.6691	-2.4173	4.0481
-1.8021	0.089	-2.0247	1.799	0.92	3.222	-0.386
-4.009	-0.793	-6.534	0.6511	-3.646	1.9583	-3.7717
0.1487	-1.0866	-0.8166	7.2932	6.5684	1.6175	1.4604
-3.3894	-6.8593	-3.1055	-2.1383	0.0311	-3.1244	-3.9694
2.9004	9.1862	5.615	2.3485	0.9297	8.7966	6.0569
-2.1123	-0.7475	-2.6773	2.774	4.0206	4.3409	0.2361
-6.9186	-7.4409	-6.1943	0.8971	-0.0781	2.1697	2.3128
-11.4297	-9.9244	-5.8217	-7.7195	-9.8678	-9.7328	-5.1836
-3.4477	-2.7411	-1.651	-4.4932	-3.5174	-2.7952	-1.7011
-3.411	-7.1503	-4.1213	-8.1897	-6.5496	-9.8806	-8.3347
-4.5826	-1.3962	-3.0174	-0.2013	-3.5334	-0.8462	-2.368
-5.9769	-5.2872	-5.5583	-2.5947	-5.8074	-5.5686	-5.7703
2.7309	3.0111	0.1958	-4.1452	2.7137	2.9692	0.1428
-2.7043	-2.5069	-3.1834	-5.9213	-2.641	-2.5427	-3.2108
-4.9278	-0.561	-1.3318	-3.5675	-4.8944	-0.6155	-1.3456
0.4136	-4.3685	-7.3294	-1.6235	0.3235	-4.4655	-7.383
-4.2054	-0.9168	-2.3539	-6.0617	-3.5058	-2.1353	-1.6356
-0.8437	-0.5554	0.5508	-1.9887	-0.8971	-0.5495	0.4649
-6.2986	-0.331	-7.1166	-2.0765	-6.3382	-0.3431	-7.0847
1.0142	-1.1694	-0.3617	-1.431	0.9634	-1.1881	-0.3221

AF4-self_p	AF4-self_n	AF4-other_	AF4-other_	AF8-self_p	AF8-self_n	AF8-other_
-3.7438	-0.9747	0.9857	1.0354	-4.6009	-0.1072	0.749
-0.8668	-1.3505	-1.6664	0.5087	-5.267	-2.596	-10.2081
-7.1351	-2.7689	-7.7499	-2.4576	-5.8863	-4.4304	-7.0359
1.8536	0.2064	0.8449	1.5215	0.1256	-1.108	-1.8611
-0.6622	0.6578	3.4843	-2.0711	-0.934	-2.7922	1.4769
-1.092	-3.5622	-1.8047	-2.3158	-0.7642	-1.059	-2.9082
-2.6771	-1.3282	0.4078	-1.2903	0.3263	-1.3372	-1.2225
2.0651	-0.0862	0.4504	-1.1244	4.0465	-0.9794	-0.0403
-2.0572	-4.5259	-2.5017	-5.1455	-4.5959	-11.678	-10.0326
4.329	3.0584	0.94	0.3279	2.3316	1.3484	-4.2906
-3.976	-4.9345	-3.9552	-3.5668	-3.5575	-2.6695	-2.4844
-0.1013	-5.097	9.6117	3.8801	2.03	-7.9057	0.5334
-2.724	3.6824	4.1974	-1.3203	-1.5557	-0.7526	-2.4303
-1.6495	-1.661	-3.3273	-2.5364	-1.582	-2.3062	-1.5441
-6.6322	-3.5224	-5.0175	-2.8446	-0.8919	0.1118	-1.8654
-5.4012	0.5265	-3.4529	-4.6145	6.6798	-2.0924	2.8288
-2.8274	-3.3833	-6.0823	-4.7672	-0.8893	-0.9429	-3.1901
-3.7031	-2.3569	-4.2071	-1.874	-1.3912	-4.073	-1.423
-0.5517	-3.1784	-3.5335	-0.1259	-4.8166	-1.2886	-1.3363
-9.7361	1.6458	-4.4581	0.199	-0.5836	5.0311	1.9392
-3.486	-2.2277	-1.5704	-1.1134	-1.3552	-1.1914	-0.472
-7.2673	-2.8067	-4.6397	-1.8273	-1.3211	-2.4783	-0.7578
-12.9069	-4.9177	-2.7854	-4.0244	-16.3449	-14.9449	-16.5613
-4.3364	-0.6573	0.5847	-0.311	-2.6025	-0.3955	-3.251
-6.6556	-5.872	0.3295	-3.0549	-4.2597	0.543	5.1658
-2.2452	-5.5343	-3.3532	-7.1045	-6.6984	-6.7446	-3.7315
3.1662	2.5037	3.0109	2.2447	3.1711	1.7913	4.747

AF8-other_r	F2-self_pos	F2-self_neg	F2-other_pc	F2-other_ne	F4-self_pos	F4-self_neg
-0.6944	-5.1054	-0.9388	-0.7014	-0.1099	-4.2468	-1.5653
1.9261	0.4307	-1.7813	-1.9657	0.4652	0.1679	-1.5232
-2.2352	-6.0331	-2.0065	-6.2038	-3.1502	-3.3497	-5.043
0.9563	-0.9315	-1.916	-0.5799	1.0631	-2.1376	-1.0394
-5.2654	-0.4135	3.0607	4.2204	-1.7243	0.0151	2.8763
0.3476	-5.005	-7.6557	-5.8423	-6.7376	-2.0283	-4.1014
0.548	-4.9538	-2.7548	-3.7487	-2.833	-3.0011	-1.2856
-0.0886	1.3262	0.047	-0.1034	-1.2206	2.4033	0.9959
-4.7856	-4.2324	-3.6351	0.5459	-10.088	0.0306	-2.5406
-0.0954	5.2874	4.6877	1.8874	1.4488	4.5488	4.1013
-3.9809	-4.2617	-5.115	-6.2836	-5.6059	-3.7237	-4.8135
15.3994	2.4796	-2.0344	5.376	1.4766	2.407	-2.805
0.416	3.0954	0.8414	0.5791	1.1944	-0.4854	2.409
-0.8442	-1.7899	-4.8772	-4.7777	-3.1119	-2.2231	-2.9923
-1.8171	-6.3033	-8.6689	-6.6842	-8.105	-6.6939	-6.0012
2.8546	-1.7887	-1.7703	-2.4269	2.5015	-4.1731	0.0408
-1.3324	-2.5867	-1.9625	-5.4375	-4.4761	-1.1009	-1.7598
-2.641	-3.5089	-4.8299	-6.8858	-4.8816	-4.1857	-3.4173
-2.7028	-8.6663	-1.9631	-3.3614	-2.0495	-2.8936	-2.1811
1.152	-2.9583	-1.078	-1.2	-0.1365	-2.0101	1.1714
-0.7244	-2.0416	-1.5867	-1.5976	-0.8332	-1.4503	-0.9861
-1.5765	-5.6157	-2.1935	-3.7807	-2.426	-3.8224	-1.1385
-19.1935	1.2889	1.7314	-0.8437	-0.3477	0.4244	0.8574
-1.0097	-3.0094	-1.0523	-0.5312	-0.2589	-2.9972	-1.0516
0.4852	-5.7321	-5.1	-0.1805	-3.8081	-4.012	-1.6884
-9.9452	-2.8728	-3.1028	-3.6038	-2.3537	-1.5505	-6.1916
1.5373	1.8354	3.175	4.072	1.5445	2.2974	4.4794

F4-other_p	F4-other_n	F6-self_po	F6-self_ne	F6-other_p	F6-other_n	F8-self_po
-0.4657	0.497	-4.3378	-0.3917	0.9212	-0.0945	-3.5851
-1.1552	1.4972	0.4136	-0.6919	-0.4886	0.9372	-0.7585
-7.3884	-0.6307	-7.3913	-4.7522	-7.9294	-1.2342	-2.1794
2.6509	1.4466	2.5406	-0.6078	1.1334	1.3092	-2.7101
3.4341	-0.7732	0.6479	1.4906	3.9938	-1.7989	-0.5917
-3.7925	-4.6092	-0.4027	-1.7275	-1.6857	-1.6446	-3.3056
-1.3898	-0.553	-0.3492	4.2759	-1.1686	-0.7106	-0.4265
0.5981	-1.4326	2.6352	0.9245	1.1836	-0.847	2.5199
-0.2492	-7.186	0.1856	-1.9873	-2.0265	-0.3616	-0.9141
-0.7629	-0.1055	3.0493	1.0095	-2.1793	-3.0897	3.2816
-5.7051	-6.2413	-4.0666	-4.797	-3.4795	-3.1445	-2.7167
8.6644	2.0934	1.3867	-4.3147	8.687	-0.0539	2.8876
1.2145	1.1933	1.6411	1.186	-2.5365	1.2515	0.7421
-2.4323	-2.9248	-1.0975	-1.241	-3.0497	-2.1782	-1.9396
-5.6249	-7.0702	-3.2279	-2.9441	-7.1775	-6.8218	-2.8185
-2.84	0.973	-6.5278	1.9939	-3.7288	-2.7436	-5.295
-4.43	-2.6618	-0.875	-1.3132	-2.7742	-1.467	2.1155
-3.7477	-4.2214	-3.7987	-2.3012	-2.859	-0.8703	-0.8616
-3.4708	-1.2785	-1.7082	-1.7794	-5.7692	-1.0777	0.0281
0.203	0.3615	-1.0014	-0.1856	0.0115	2.1681	-1.9797
-0.9433	-0.5164	-2.7606	-1.8015	-1.2197	-0.143	-0.8346
-1.6993	-1.1272	-1.7767	-1.0456	-0.8432	-1.5275	-1.117
-2.0525	0.5176	-3.4728	-1.0219	-6.8244	-4.5829	-4.2155
-0.5405	-0.2099	-3.0797	0.0244	-0.2501	0.7872	-3.5678
1.1029	-1.9774	-2.2545	-0.5279	2.2131	-0.103	-0.9391
-6.2732	-3.4209	-9.579	-7.5224	-0.6195	-1.6568	-0.6006
4.4033	3.0561	2.513	4.066	3.8946	3.3887	1.932

F8-self_neg	F8-other_po	F8-other_ne	FC2-self_po	FC2-self_ne	FC2-other_p	FC2-other_n
-1.3428	1.1075	0.2815	-2.8319	1.0025	-1.117	-1.4259
-1.9442	-2.7654	0.9307	2.98	0.4461	-0.0561	1.5834
-3.809	-7.4693	-3.4661	-4.5783	0.791	-4.6061	-1.3209
-1.0594	-2.0612	-0.2834	-0.9649	-2.036	-0.4614	1.349
-0.1056	2.6799	-2.3533	1.9025	2.4074	4.2771	1.4225
-5.4414	-1.0182	1.6603	-5.379	-8.267	-5.3956	-5.4941
4.3115	-1.1427	-0.7395	-4.3315	-3.2564	-3.1628	-3.4789
1.3101	1.0696	-0.889	2.3943	1.9626	2.8259	-0.2254
-1.8777	-4.5825	3.1919	-2.4841	-2.6002	1.6864	-8.0037
6.3956	-0.6377	0.3377	6.9393	5.8863	3.4981	2.977
-3.4425	-1.8254	-3.1009	-3.7548	-4.9767	-3.3128	-3.2711
-3.1443	2.9307	2.0061	2.5917	1.0617	4.9753	1.6443
-1.4582	-4.9418	-0.4161	3.659	3.5485	2.4455	1.885
-2.2093	-2.646	0.289	-2.9154	-3.7742	-4.1297	-3.7135
-0.1881	-5.0325	-5.5713	-4.7166	-6.3811	-4.3512	-6.4415
2.507	-2.6007	-1.381	-0.3222	0.5249	0.3312	4.2603
-0.2806	-4.6551	2.6385	0.275	0.9035	-1.4125	-1.9672
-1.3137	-1.0566	-0.2562	-4.2314	-3.4764	-6.3029	-5.0438
-0.7965	-6.3685	0.5655	-7.6246	-0.3283	-4.5034	-0.4524
0.5213	1.0035	1.4334	-2.8859	-1.182	-1.3883	0.3892
-1.0034	-0.8526	-0.4443	-0.929	-0.327	0.2945	1.9966
0.3682	-1.4211	-1.522	-0.9681	0.2812	1.1617	0.138
-3.1241	-6.4318	-6.4989	0.2508	1.1664	-2.1471	-1.2346
0.1835	0.869	0.6867	-1.7279	-0.0695	2.0832	1.325
-0.4754	2.0491	-0.1804	-4.2036	-2.9149	0.9235	-2.1603
-4.7233	-1.6354	3.003	-3.2623	-2.9728	-0.683	0.0182
3.3962	3.3907	2.6646	0.306	2.7635	4.0959	0.5777

FC4-self_p(	FC4-self_n(	FC4-other_I	FC4-other_I	FC6-self_p(	FC6-self_n(	FC6-other_I
-3.0934	-0.4889	0.7281	0.7068	-3.4834	-0.0118	1.0669
1.1401	-0.1801	0.0835	1.3505	0.4522	-2.064	-1.6304
-3.245	-1.3027	-5.0636	-0.8907	-2.6615	-1.6236	-4.8402
-0.8909	-0.9042	-0.1407	1.9498	-2.183	0.3234	-1.2877
2.2313	5.5055	4.4076	-0.1328	0.686	2.8476	6.1891
-2.7578	-4.3944	-4.6542	-4.4616	0.1859	-2.3726	-3.0489
-1.7363	1.9459	-1.8733	-3.9035	2.397	3.1336	0.7664
3.441	2.1726	2.8685	1.1114	3.21	2.3223	2.2041
-2.2464	-1.8145	1.1692	-12.0553	2.3697	0.8806	1.8662
5.0893	4.7035	-0.2908	2.4769	5.1	5.8455	1.4693
-2.9569	-2.3124	-3.3044	-3.9226	-3.0746	-3.4231	-3.3604
3.5142	0.6667	2.5561	2.9172	-0.7531	-0.087	8.7384
1.8352	3.47	0.9996	0.9348	-0.7576	0.4507	0.3155
-2.4507	-2.7998	-2.9956	-3.1872	-2.3441	-1.6223	-1.734
-2.5977	-5.8408	-5.43	-8.5774	-0.6298	0.2009	-3.9676
-0.4908	0.4235	1.4364	3.9781	-2.533	2.552	-0.6718
-0.5308	0.8671	-1.4347	-2.0719	0.2764	-0.1008	-1.5477
-6.2119	-1.9243	-3.7121	-4.1194	-3.0766	-1.3788	-3.7038
-3.7334	-3.7298	-1.5071	-3.0764	-4.2755	-3.6537	-1.6774
-1.6584	-0.7808	-0.2262	0.0255	-1.169	0.9371	-0.1181
-1.2875	0.2242	-0.0487	1.893	-0.505	-0.067	0.3317
-0.2239	1.5507	1.3172	0.1903	-0.7615	-0.1711	0.2952
0.423	3.5537	-2.7439	1.1458	-2.055	1.2115	-5.443
-2.2593	1.5536	0.82	0.6811	-1.8775	0.0064	1.986
-1.724	-0.8971	2.4722	-0.7821	-0.6977	-0.1638	3.0304
3.3041	-3.4018	1.6859	-0.1291	-0.3231	-0.9539	-1.1342
2.2971	3.4804	4.3586	2.3173	2.367	3.9234	3.9987

FC6-other_r	FT8-self_p	FT8-self_n	FT8-other_	FT8-other_r	C2-self_po	C2-self_ne
0.2336	-3.6741	0.5464	1.8851	-0.4882	-3.0706	1.3322
0.0126	0.0516	-1.6322	-1.1516	-0.3727	4.871	1.3316
-1.9907	-0.331	-3.2197	-5.2797	-1.7266	-1.6645	3.2806
0.4381	-2.4193	-1.0317	-1.6431	-0.663	-0.3018	0.039
1.1643	2.266	0.3914	2.1314	1.0292	3.522	6.027
-1.1477	-1.5067	-3.4495	-2.9261	0.4758	-2.4658	-5.0042
-2.4929	0.0818	3.8203	-0.075	-2.5069	-3.377	-1.4642
0.551	3.2144	2.0787	1.8539	0.0386	4.2168	3.7815
-0.4898	0.8242	-1.0669	-0.6311	1.8085	0.972	0.0572
2.0544	1.6667	1.7423	-1.8456	1.8235	12.0985	4.7971
-3.0018	-2.4132	-2.1186	-1.6136	-1.1537	-2.1829	-2.7686
2.089	-0.8592	-1.9655	3.7796	-0.6683	3.5425	2.3956
2.9325	2.5922	0.326	-6.0892	-0.5801	5.842	4.65
-1.4586	-0.7715	-1.5365	-2.3151	-1.451	-1.558	-2.7929
-3.3015	-0.0276	4.9162	-0.5952	0.3821	2.0302	-8.297
-1.185	1.7665	1.5374	1.4419	4.8539	1.9254	1.7783
-0.6439	3.7984	1.0034	1.9245	0.7327	2.3188	2.3451
-0.5837	-2.6899	-1.304	-2.556	-3.0239	-3.5696	-1.1254
-1.2917	-3.7417	-3.3968	-2.5972	-0.1451	-3.5907	-0.1476
1.0457	-1.0801	-0.4362	-0.3846	0.9204	-2.0819	-1.4411
1.5679	-0.2175	0.0489	0.0478	0.6147	0.9892	0.5891
-1.0345	-1.9078	-0.3996	-0.2457	-1.2497	1.6286	3.5462
-2.4897	-1.0775	-5.0654	-0.9801	0.46	3.5455	3.0474
2.5885	-2.2008	-0.7463	2.5992	1.2447	-0.4667	3.3737
-1.215	-0.4569	0.9518	3.4986	1.981	0.0864	0.4886
-1.0078	-1.4676	-0.7562	-2.9271	-0.5367	0.117	-0.0807
2.8462	1.5802	3.3148	3.101	2.4837	0.1363	3.6885

C2-other_p(	C2-other_n(	C4-self_po:	C4-self_ne;	C4-other_p(	C4-other_n(	C6-self_po:
0.225	0.6784	-3.4678	-0.0854	0.7619	0.8314	-2.7474
2.3801	1.9614	1.3109	0.9528	1.3827	1.5959	0.3024
-2.0282	1.0158	-0.8706	2.2279	-2.0392	1.4425	-1.7089
0.3248	1.9651	-0.5964	-0.2345	-0.4311	1.593	-1.7172
6.063	1.1982	4.6195	6.3685	7.6031	1.273	4.1263
-4.4593	-4.5887	-2.5285	-2.3892	-2.9318	-1.7661	-1.1098
-1.5559	-0.8087	-1.7554	0.4723	0.1361	-0.2658	-0.1816
4.3736	2.6413	4.941	4.2239	5.4163	3.0963	4.7875
1.9397	-1.7102	0.9122	2.9472	3.283	-1.6005	2.857
4.4041	2.4276	7.1195	6.6271	3.5125	3.0049	5.9251
-2.4871	-0.4025	-2.6456	-2.2187	-2.4929	-1.294	-1.7437
6.7074	2.0547	2.4903	-0.1009	6.5409	3.821	2.9538
3.9085	3.2398	4.2595	3.259	2.0169	3.2124	-0.0898
-2.5536	-2.2899	0.2883	-2.5871	-2.59	-1.3836	-0.3773
-2.7535	-5.4003	-0.5094	-1.4349	-2.1983	-5.1848	2.0027
4.7125	5.9876	2.9641	3.9765	4.7905	6.7419	-4.1779
1.8096	-1.0699	2.5812	2.0306	2.2993	-0.5248	0.7823
-2.2898	-2.5313	-2.3811	-1.1231	-1.5272	-2.0009	-1.4141
-4.0199	1.6852	-3.0941	-2.1105	-1.414	0.9849	-3.6637
-0.113	1.6785	-0.8115	0.776	-0.1155	1.72	-0.5122
2.3658	3.7626	1.5552	1.5871	2.4317	3.4172	1.1835
5.2667	2.6493	1.5792	2.8742	4.9594	1.9729	0.96
0.6652	0.3212	2.6167	0.415	-3.31	-1.2266	-3.0482
2.1483	2.6702	-1.5243	2.0158	1.5188	1.6672	-2.2043
4.3707	0.5513	1.7539	0.7771	4.2219	1.4471	0.6093
1.3814	0.461	1.0064	-0.841	0.3093	-1.2586	1.363
3.5375	0.2473	1.6609	3.1599	3.9976	0.534	1.2937

C6-self_neg	C6-other_po	C6-other_ne	T8-self_pos	T8-self_neg	T8-other_po	T8-other_ne
0.4655	0.8455	0.9268	-3.1894	-0.447	1.6441	0.3058
-1.2712	-0.7073	0.1191	4.5046	0.6944	3.994	0.4606
0.8054	-3.6132	-1.2446	-0.2199	-1.3914	-4.0898	-0.9424
-1.2891	-1.4918	0.8873	-1.2511	-0.7202	-1.026	0.0904
4.8665	6.0792	0.5061	3.0703	0.4405	3.4873	-0.1779
-1.2497	-1.1221	-0.1395	-1.3463	-3.1798	-2.64	-2.6206
1.2577	1.6026	0.3934	-0.4858	3.5402	3.0888	-0.063
3.601	2.628	0.7863	3.54	3.0714	3.195	1.3879
2.3903	1.684	2.2221	4.7241	5.532	-0.534	2.2868
5.8291	1.9521	3.634	3.5202	3.6167	0.7655	3.0349
-1.4885	-2.2168	-1.6199	-1.6259	-0.2458	-0.6822	-0.646
1.8508	4.6729	1.8446	0.3883	-0.5805	3.4749	1.637
0.709	1.4404	1.3911	1.9549	0.5501	0.0827	1.6914
-1.657	-2.4867	-1.1628	-3.0222	-1.7995	-0.5698	-2.0359
1.4304	-1.4819	-1.7576	1.0922	1.7138	0.9436	3.3536
6.7546	-0.5887	-0.1447	-1.5327	0.9683	0.2671	-2.5852
1.0835	0.0928	-0.1514	4.1864	1.7311	5.3097	5.044
-0.6601	-0.8606	-0.6666	1.6129	-1.5689	-3.4096	-2.9269
-3.6645	-4.032	1.6024	-5.3806	3.0037	-2.3103	2.1865
0.1232	-1.6937	1.579	-1.6901	-1.2125	-1.613	-0.1377
1.592	1.5381	2.7997	0.8825	0.4107	1.0004	1.5917
0.1927	2.8444	-0.5268	-0.2465	-0.0487	0.9319	-0.9517
-1.9527	-5.1361	3.9369	2.5959	3.0498	-3.8482	-2.0141
0.6493	0.3996	-0.1356	-1.4312	1.0902	1.106	1.1621
0.3861	4.9427	0.0559	0.928	1.3221	4.475	1.883
-0.3836	-0.0682	-0.5463	1.1073	-0.1869	-0.8667	-0.2812
2.879	3.1347	1.6514	0.8098	2.1948	2.7689	1.9652

Cz-self_po	Cz-self_ne	Cz-other_p	Cz-other_n	CP2-self_p	CP2-self_n	CP2-other_
0.5442	-0.7234	3.7991	8.3439	-3.1581	2.1851	0.1134
3.1506	1.7891	1.4876	0.9993	3.2481	3.0177	3.5537
-1.4349	3.3436	-2.6998	0.4194	1.8699	5.8948	1.9091
5.3789	0.0003	6.9794	0.4711	0.8727	2.7312	1.3375
2.8136	6.2458	5.3566	0.8745	5.6742	8.1284	8.2967
-4.459	-7.6571	-5.3399	-7.081	0.3886	-2.1091	-1.9574
-4.2476	-0.8825	-1.8313	-1.5041	-0.9913	1.5553	1.4863
3.1454	2.9744	3.7768	1.5389	6.2292	6.3779	6.7692
0.3116	0.5559	1.8743	-4.5902	4.9118	4.718	5.7721
9.3116	6.5702	6.7619	3.9917	11.4478	7.9285	6.1583
-2.597	-3.9184	-2.3853	-1.3104	-1.0515	-2.0323	0.577
1.3613	0.8284	7.6748	1.6817	1.6083	3.6153	7.2055
6.5092	5.3488	4.3315	2.8395	7.9601	5.1018	5.7021
-1.1482	-3.457	-2.8786	-1.2329	0.395	-0.424	2.4528
-3.3199	-5.6162	-2.4301	-5.7866	1.9633	0.5721	-0.1291
1.9281	2.7512	4.2522	5.4522	5.8546	6.7116	7.3935
1.233	1.3843	2.8534	-3.9042	4.327	3.7011	3.2515
-3.9872	-2.0218	-2.7543	-3.7047	-1.0334	1.2265	0.0542
-2.9374	-3.57	-0.7596	-0.8446	-1.1875	-0.4171	-1.8143
-1.7613	-1.1395	-0.15	-0.1557	0.3083	-0.1419	1.646
0.0778	0.7488	1.7364	2.5162	2.9264	3.0057	4.23
-0.039	2.2669	3.6906	2.4242	3.5087	6.1329	8.967
3.3129	2.649	-0.7041	-0.3276	4.5651	5.9056	3.2158
0.2215	2.8562	1.585	2.5951	0.8345	3.6545	2.9948
-1.5214	-0.682	2.9405	-1.1508	5.6119	4.4667	6.1172
1.259	-1.3333	0.8174	-1.5368	-0.7573	0.4852	2.7224
0.0873	2.6465	3.8137	-0.4742	1.5258	3.056	4.7914

CP2-other_r	CP4-self_p	CP4-self_n	CP4-other_p	CP4-other_r	CP6-self_p	CP6-self_n
1.1399	2.0055	-3.183	2.434	4.1596	-0.9622	1.2779
2.0074	3.0824	2.4523	2.4916	2.7391	1.6871	0.0754
5.1449	1.4595	4.8538	1.1431	3.3949	0.0267	1.9373
2.5008	0.2045	2.5878	0.9912	2.739	-5.4341	6.9734
2.4955	5.6395	3.1153	7.1733	0.9059	6.1596	3.9718
-1.7719	1.1949	-1.6032	-1.1373	-1.0635	-1.6474	-1.222
1.901	-0.0009	2.3153	1.5898	1.9094	1.161	1.4102
4.4449	7.3306	7.0274	7.3556	4.9682	6.8931	6.2907
2.9223	3.7963	4.5102	4.6958	2.8493	4.2852	6.3916
3.449	6.5022	7.3733	-0.9547	5.9591	6.7994	3.8317
2.1259	-0.8288	0.2045	-1.0442	-0.556	-0.9216	-1.2188
2.6009	3.1959	2.5388	3.8318	1.9829	2.3553	1.386
4.1079	3.7465	3.584	4.0584	4.6736	1.5631	2.1469
1.5017	-0.0652	-0.8211	-0.2962	-0.3159	-0.41	-0.0005
-3.5284	3.5105	1.8864	1.0887	-1.1372	3.1149	4.9421
8.0271	5.4846	5.8921	7.1984	6.3741	1.492	10.631
0.953	4.9112	3.7396	3.3991	1.1456	2.9555	2.0852
-0.3912	-0.3441	1.2232	0.1884	0.6581	-0.382	0.2391
2.2945	-2.0478	-1.4268	-1.885	2.4502	-2.1139	-3.7824
2.3953	0.721	2.0791	0.9387	2.7621	0.0602	1.0215
4.6789	3.1474	3.2013	3.9783	4.5026	2.9974	2.7801
6.482	3.21	4.7821	6.7523	2.9128	4.2626	4.572
2.1844	3.6837	5.5878	0.647	0.6663	6.5588	5.3038
3.5477	-1.273	4.0446	2.8584	1.6961	0.2968	1.5752
4.0222	5.0101	3.7363	6.0599	2.7772	4.3114	0.9194
1.9191	1.9951	0.5884	3.8377	1.0304	1.4719	-0.7322
-0.2404	1.8032	3.4596	3.3309	0.5084	0.4955	2.4623

CP6-other_I	CP6-other_I	TP8-self_p	TP8-self_n	TP8-other_I	TP8-other_I	P2-self_pos
1.1867	3.606	-0.2526	1.491	1.6183	1.5071	1.0075
1.3151	1.1187	2.5273	1.8348	0.9851	1.8568	10.1548
-1.453	2.7903	1.5897	1.3782	-1.3514	0.1414	5.2385
2.9838	0.1479	-0.7042	0.064	-0.1165	0.7964	0.9067
6.5309	1.8404	4.5538	3.2832	5.3138	-0.0405	6.2287
-0.8716	1.6948	-0.8084	-0.8203	-0.2919	1.3323	2.076
2.9186	3.0978	1.5138	1.6494	3.4647	3.1986	3.621
6.0603	3.8124	4.5996	4.5101	4.0256	2.4735	8.4015
1.7822	2.6674	3.0323	4.7704	-1.1701	1.9688	7.3846
5.8879	6.083	3.9235	2.1625	0.1075	2.1074	10.8814
-0.7167	1.3938	-0.4436	0.4319	-1.9328	1.234	1.3629
3.5912	2.5671	-1.2766	-0.6939	4.8725	0.5194	1.5409
2.0882	5.838	0.011	3.6658	2.2385	2.8689	7.9595
1.6406	-0.1983	-0.4912	-0.2191	0.0124	-0.8462	0.6579
-1.367	-2.0319	-0.8368	4.7356	-0.0027	-0.5352	4.6058
6.5666	9.2583	-0.1976	1.5295	4.8626	-1.4872	8.3521
3.4539	1.6089	3.4059	3.23	3.439	2.1037	5.6252
0.2807	-1.2324	1.2205	3.4379	1.5556	1.9543	1.9107
-2.0108	-0.2626	-0.6324	1.6836	3.0745	-0.9956	-1.6345
0.5332	1.7024	-0.0353	-0.089	1.4941	0.993	1.139
2.586	3.3586	1.2923	1.4156	0.9348	1.8646	5.7636
2.2483	1.5007	0.8162	0.8007	2.0386	-0.7502	7.6824
1.9944	2.9297	3.7144	3.5439	0.4213	2.3575	5.1941
0.9108	1.4948	-2.3236	2.2116	0.0968	1.204	0.7516
5.3049	1.8985	1.2355	2.8809	5.5049	1.9379	8.7834
3.1815	3.6267	1.5002	1.4589	-1.4681	2.1108	4.9716
2.1682	-0.9353	-1.1752	0.8811	1.0439	0.1657	1.3713

P2-self_neg	P2-other_po	P2-other_ne	P4-self_pos	P4-self_neg	P4-other_po	P4-other_ne
2.5906	1.6787	2.8383	2.0145	3.9492	3.3885	3.4801
8.4549	8.0883	2.33	2.7626	7.6544	3.355	3.2641
8.7842	2.5059	6.1849	3.9893	7.8044	1.6092	4.6693
7.8322	4.912	1.8408	6.3938	2.6369	1.8476	7.2132
7.7812	8.9388	2.2252	6.2611	5.2434	8.3443	0.8801
0.6405	0.796	2.9483	3.6961	-1.6277	1.861	6.5225
2.1195	6.8314	0.1213	4.3358	2.5136	3.7527	4.9641
8.7275	9.0819	6.986	8.7735	8.8756	9.4192	8.0648
6.2352	7.809	4.5793	5.8842	5.5687	6.7454	4.1084
9.1008	6.3362	7.573	12.2241	10.2268	7.8782	8.7393
0.8053	1.5819	2.0075	1.0227	1.0825	1.1714	2.6649
3.7237	5.875	2.8765	-1.182	-0.1953	0.4909	4.3209
7.2252	7.2089	5.8473	10.9726	5.6905	5.6593	4.549
-0.1779	1.1221	1.5523	0.6904	0.4119	2.5949	1.1304
2.8685	-0.1345	-4.2833	4.633	4.2529	-0.5058	-4.8712
10.1361	11.8746	9.4673	7.578	10.9233	10.6531	6.8994
4.4477	4.8172	1.7169	7.2747	4.8127	7.9727	4.3597
3.2085	1.0299	1.3984	3.4799	3.4844	0.8526	-2.9139
-1.3243	0.288	4.7972	0.909	-1.5413	2.1739	5.1874
1.4965	1.7619	3.0431	1.3379	2.0411	0.9504	3.4967
5.3327	6.2519	6.6875	4.6626	5.298	5.4372	6.1828
8.6499	11.2354	5.5945	6.3359	7.5976	11.2666	5.2389
8.7139	5.0406	3.4472	3.4395	7.359	0.0762	4.0274
3.5492	3.094	3.6306	-0.1347	4.813	3.254	2.116
4.0834	7.3934	4.9932	8.9409	5.3895	8.1962	5.625
2.636	7.2014	6.8027	4.7728	0.2367	5.9757	4.4328
2.5561	3.8405	0.6153	1.5246	2.4991	2.2711	0.5802

P6-self_po:	P6-self_ne{	P6-other_p(	P6-other_n(	P8-self_po:	P8-self_ne{	P8-other_p(
1.2918	2.8668	2.4983	2.5606	1.2983	2.5988	2.229
4.0355	4.2739	2.1405	3.2615	4.2066	3.878	3.7667
0.1383	5.2549	0.8376	3.9502	3.0819	3.5411	0.2534
-0.0472	1.7212	0.7733	1.7922	-0.3859	0.9846	-0.0122
6.9194	5.9942	8.4126	1.1978	5.4713	3.333	6.1767
2.7241	-0.5485	3.478	5.861	3.7033	0.65	0.7223
1.2408	2.9839	2.5683	5.4922	3.018	-0.1288	4.5103
8.0002	8.1546	7.8375	5.8042	5.8622	5.8476	5.8065
6.1666	5.9972	4.4953	3.7958	5.4378	5.9491	0.8058
9.3606	7.729	8.2406	7.5819	3.5582	3.5785	4.2523
0.285	0.4431	0.6915	1.5557	-0.9829	0.3863	1.0724
-4.3115	-2.8063	1.8574	4.9115	-5.7827	-1.6456	-2.1901
5.7808	3.6638	2.849	-0.8291	6.2974	2.0984	2.7384
0.3848	0.586	1.6637	2.9679	-0.4725	-0.0203	1.6103
5.9788	4.7818	0.0027	-3.5928	2.1988	2.9781	-1.3501
7.282	13.8616	12.0964	10.0654	3.0425	10.7948	8.8856
4.9997	3.227	4.8659	2.5711	-0.6431	1.1188	2.5864
-1.1609	-0.3566	1.5759	2.7511	2.0044	0.5079	2.6801
3.3509	-1.5177	2.2676	4.189	0.8381	0.4979	2.1011
1.1058	2.1018	2.281	3.302	0.2565	1.4656	1.7497
3.8701	5.2178	4.9586	5.826	3.7496	3.4736	2.6502
4.1676	4.4133	3.9865	1.5638	1.3301	3.5936	3.4951
1.4232	8.0578	-1.6383	4.0761	8.4484	4.6277	3.2544
-1.337	0.5417	-0.0661	1.802	-0.5873	3.7205	0.629
5.441	2.4141	2.448	5.7939	3.179	0.6182	2.9336
3.143	-2.1904	1.8454	3.8567	2.2021	2.3395	2.0064
-0.1063	0.5895	2.0516	-0.435	-2.3798	-0.6672	-0.3557

P8-other_ne	Pz-self_pos	Pz-self_neg	Pz-other_pc	Pz-other_ne	P04-self_pc	P04-self_ne
1.4617	0.5023	2.4286	2.9774	2.9552	2.0617	4.0526
2.1469	4.0519	5.0435	3.1266	3.4911	3.2053	3.4821
1.5449	4.3674	9.5208	4.6109	6.6674	4.9976	8.2266
0.686	2.3978	3.7018	2.9401	4.0211	-1.1461	4.8658
-0.0418	7.4109	8.627	9.3358	2.9541	6.0645	3.1419
4.2963	3.2798	0.8233	0.9641	1.4418	2.9912	0.6136
5.5554	1.3905	3.781	3.7733	1.7617	6.0099	2.8519
4.533	8.0773	8.6498	8.658	6.161	6.5216	6.6937
3.6469	7.1833	7.6512	8.6298	5.7602	8.6366	7.9422
1.4611	12.242	9.8694	9.7709	3.6673	8.3938	7.7787
1.4733	0.2664	1.2622	0.2269	1.6127	2.3889	1.825
4.6483	-1.5388	2.3182	5.784	4.8481	-2.2464	0.9158
1.5376	5.2558	7.5328	4.3676	8.6302	8.7457	4.4224
0.2331	1.3678	0.1422	0.6807	2.11	0.093	1.0945
-3.5931	3.235	2.0201	1.6981	-2.3644	1.4989	0.08
5.5924	8.6808	9.4871	12.5795	10.4148	7.0716	14.538
2.2679	6.2425	4.1519	8.1239	2.8701	8.8026	5.7677
0.2044	1.7063	3.9422	2.4926	1.7792	1.9919	3.48
2.9249	-0.2944	1.8527	2.0219	3.2293	1.343	1.0383
1.4638	0.3951	2.1586	2.0715	4.1875	2.5054	3.6937
3.7783	5.1621	4.3475	5.6724	6.2193	6.1521	6.5078
-0.1682	9.8114	10.6201	13.3555	8.358	4.463	4.913
2.2389	5.5068	7.1306	5.3945	4.4966	1.9368	5.9247
3.1198	2.9847	4.5234	4.1197	3.0284	2.0677	2.2281
2.7324	8.5844	5.9483	7.2895	4.7988	8.4085	3.961
-0.4389	5.6043	5.0567	4.4815	0.2244	2.7429	2.5434
-1.6372	0.8149	2.8863	3.0854	0.8263	1.4605	0.9018

P04-other_p	P04-other_n	P08-self_p	P08-self_n	P08-other_p	P08-other_n	P0z-self_p
3.4739	3.5297	1.6858	4.8778	2.535	2.0777	4.0829
5.6846	5.3405	3.5081	4.6804	4.3738	2.1352	3.5596
3.2244	5.681	5.1976	4.5057	3.4795	3.9235	6.0346
-0.3065	4.1176	-0.8394	1.2903	-0.255	0.4093	2.546
7.3945	0.0359	6.2545	2.0415	4.7465	-0.408	6.7342
1.8605	6.9042	0.6871	-2.0724	1.9817	4.585	1.5829
2.5883	4.0333	6.0433	3.8193	3.8479	4.6946	3.8414
7.1914	4.7771	5.9025	5.9458	6.5946	4.81	6.5542
7.6164	4.9751	5.9053	5.0589	5.4801	3.765	8.0972
8.216	6.3167	5.7292	1.8671	1.5451	6.7162	7.8463
1.9641	1.9438	0.5347	2.0627	1.6356	3.9768	1.1418
-8.2154	5.1785	-3.1326	-0.8376	-9.7542	1.1357	-3.0687
2.7325	3.4801	5.6722	2.6312	2.0226	1.234	8.0282
1.915	1.6846	-0.2201	-0.3079	1.7322	1.2462	1.0901
-1.9508	-4.1225	-0.4909	2.2006	-2.4218	-3.115	2.8973
12.7628	9.3375	6.1433	3.21	6.5186	10.02	9.6882
9.4731	5.0904	5.7024	5.6293	5.3678	4.0111	8.1579
2.5788	2.8146	1.2325	0.3197	-1.0501	1.2317	2.1548
2.9468	3.7271	-1.5896	1.7117	2.4067	1.1998	4.6431
3.3059	3.143	1.0514	2.1249	2.2153	3.1217	3.9538
5.2246	6.3928	3.7575	4.1801	3.7925	4.5305	6.8605
6.8926	2.0254	1.7072	3.1152	4.1561	1.4738	8.5652
2.2715	4.155	1.2603	3.9512	0.8066	2.2397	5.8153
3.0016	1.4663	-0.3782	0.5906	1.7103	-0.0941	0.7952
5.1911	3.1896	5.4904	1.1599	2.7642	1.2004	8.8683
0.4766	3.1906	-0.8783	5.1172	0.4444	6.0296	3.7908
3.8089	0.4817	-0.013	-2.6257	-2.5986	-2.5404	1.0407

P0z-self_ne	P0z-other_p	P0z-other_n	02-self_pos	02-self_neg	02-other_po	02-other_ne
4.4174	0.3614	5.2123	2.9476	9.2671	2.766	2.2561
4.1286	5.1368	3.6982	3.6467	3.8992	3.148	2.9241
11.5433	6.1315	6.3547	5.2112	8.1241	2.0559	4.4017
4.6375	3.5511	2.6966	-0.1383	2.0765	-2.0435	0.5314
6.1163	7.7677	0.9747	4.0785	3.9654	4.715	-0.8062
3.6657	2.6482	3.4745	1.636	-0.9592	0.1523	5.758
3.1382	4.4865	5.4465	6.0691	2.8448	2.4845	4.0049
7.0721	6.701	4.6371	6.422	6.6563	7.1027	4.6823
9.9216	5.0078	8.5995	7.4012	8.5681	6.0897	5.1865
9.2936	8.2842	5.8284	3.8514	3.6566	1.7614	4.9147
2.7728	1.6171	1.8478	1.2251	0.1705	1.0976	3.0111
1.3536	-7.2191	1.6084	-3.9153	-0.8242	-12.9405	3.1757
5.8975	1.3087	3.3545	6.3331	2.1907	7.045	-0.2736
0.7313	1.3954	2.84	-1.3304	-1.4877	1.1769	-0.1006
0.8729	1.7339	-2.9495	1.0743	-0.7043	-1.828	-4.821
10.3953	13.2026	4.4448	5.2019	6.9187	7.767	2.2563
5.5786	8.7657	4.7405	9.4731	5.0331	7.1668	5.2891
5.0927	2.0394	3.2767	0.6951	1.5958	1.2051	1.1822
0.5835	-0.0657	6.1386	0.4915	-0.5915	2.072	2.3793
3.5127	2.551	3.0671	2.6879	3.2856	2.3114	3.5238
5.211	6.2646	5.8891	6.161	6.1496	5.0504	6.1092
9.7035	11.3729	6.3538	4.3334	4.9585	6.9019	1.9796
7.8058	6.5314	4.8292	1.211	5.8725	2.6248	3.8429
3.4394	1.9273	3.2277	-0.5476	0.9246	2.1478	0.7534
4.1658	7.5918	4.9311	-7.2808	-9.0147	2.8293	1.2678
1.7533	3.1954	2.1896	3.4101	2.9717	4.3531	3.8887
2.9832	3.4968	1.3435	-1.2038	0.1376	0.0442	-0.6351

VEOG-self_	VEOG-self_	VEOG-other_	VEOG-other_negative
41.0972	19.2397	32.63	45.6116
-5.34	-2.5194	-1.901	0.2818
24.7666	26.3054	24.2261	39.8355
-2.1609	-0.1553	-20.0388	-1.0441
-0.5412	-15.8065	-7.2547	-1.3004
14.7095	20.7612	20.022	16.7123
10.0057	2.3221	8.7966	3.2647
51.5908	38.7855	50.2414	56.3784
7.0472	0.505	-9.3059	5.5279
2.0337	-7.9108	-1.3476	-7.0958
3.9925	13.9349	6.2149	1.1276
3.4596	16.7554	-5.4474	-3.902
-0.2511	-3.1581	2.9183	0.9259
34.8215	21.1754	28.4375	26.487
19.5914	14.7797	2.1351	16.3903
10.8238	25.0984	35.3944	-12.4035
2.2028	-0.7324	2.3164	-1.8581
7.6652	5.0581	24.1949	4.9011
19.0239	-18.6555	3.8179	24.5612
1.8909	12.0993	-4.0335	3.2044
-4.2914	0.3721	-2.4314	-3.3183
5.0303	17.5158	20.2294	3.6043
3.9254	6.2079	20.9467	30.3182
-8.3861	-9.5742	-3.6556	-5.2136
5.7605	-3.4062	1.1783	-0.2416
2.2144	6.4776	6.2985	6.6847
1.622	2.6019	1.3038	-0.2176
